# Pressure-induced commensurate stacking of graphene on boron nitride

**DOI:** 10.1038/ncomms13168

**Published:** 2016-10-20

**Authors:** Matthew Yankowitz, K. Watanabe, T. Taniguchi, Pablo San-Jose, Brian J. LeRoy

**Affiliations:** 1 Physics Department, University of Arizona, Tucson, Arizona 85721, USA; 2 National Institute for Materials Science, 1-1 Namiki, Tsukuba 305-0044, Japan; 3 Instituto de Ciencia de Materiales de Madrid (ICMM-CSIC), Cantoblanco, 28049 Madrid, Spain

## Abstract

Combining atomically-thin van der Waals materials into heterostructures provides a powerful path towards the creation of designer electronic devices. The interaction strength between neighbouring layers, most easily controlled through their interlayer separation, can have significant influence on the electronic properties of these composite materials. Here, we demonstrate unprecedented control over interlayer interactions by locally modifying the interlayer separation between graphene and boron nitride, which we achieve by applying pressure with a scanning tunnelling microscopy tip. For the special case of aligned or nearly-aligned graphene on boron nitride, the graphene lattice can stretch and compress locally to compensate for the slight lattice mismatch between the two materials. We find that modifying the interlayer separation directly tunes the lattice strain and induces commensurate stacking underneath the tip. Our results motivate future studies tailoring the electronic properties of van der Waals heterostructures by controlling the interlayer separation of the entire device using hydrostatic pressure.

## Abstract (web summary)

Van der Waals heterostructures enable fabrication of materials with engineered functionalities. Here, the authors demonstrate precise control over the interaction between layers by application of pressure with a scanning tunnelling microscopy tip.

## Graphical abstract

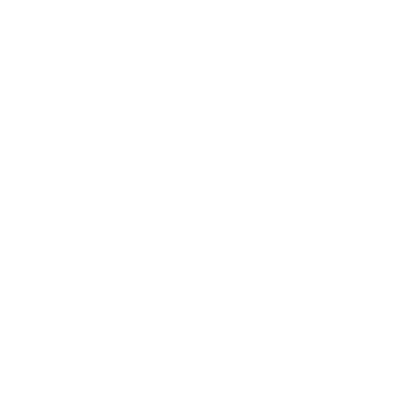

The electronic properties of heterostructures of van der Waals (vdW) materials are expected to depend on the exact nature of the interactions between the composite layers. Previous work has focused on controlling the properties of these systems through the choice and ordering of the materials in the heterostructure, as well as the rotational alignment between layers^1^, but little has been done to explore the interlayer separation degree of freedom. In bilayer graphene, for example, the electronic coupling between the two layers depends exponentially on their separation^2^, controlling the effective mass of the charge carriers and the magnitude of the field-tunable band gap^3^. For graphene on atomically-heavy materials, such as WSe_2_ or topological insulators, the strong substrate spin–orbit interaction (SOI) is predicted to strongly enhance the SOI in the graphene and possibly induce topologically non-trivial insulating states^4^,^5^. The predicted magnitude of the SOI in the graphene also depends critically on the interlayer separation in such structures. Less immediately apparent, modifying the interlayer separation through pressure can also induce a commensurate match between two crystals with slight lattice mismatch at equilibrium.

Graphene on hexagonal boron nitride (hBN) is an excellent testbed for this effect, as a long-wavelength periodic interaction emerges when the two crystals are in near-rotational alignment due to their small lattice mismatch (*δ*∼1.8%) (refs 6, 7, 8). This moiré pattern spatially modulates both the electronic coupling, and the vdW adhesion between the graphene and hBN lattices. The periodic modulation of the electronic potential leads to secondary Dirac cones in the graphene spectrum^9^, while the modulation of the adhesion potential is expected to produce periodic in-plane strains of the graphene lattice. The latter arise because the adhesion potential is stronger for carbon–boron (CB) stacking than for any other lattice alignment. As a result, the graphene lattice expands locally around CB-stacked regions to increase the area of this favored stacking. This occurs at the expense of other stacking configurations, so that the total adhesion plus elastic energies is minimized^10^. A small out-of-plane lattice corrugation matching the moiré also develops to minimize the total potential energy of the system^11^,^12^,^13^ (Supplementary Note 4; Supplementary Fig. 7). Small electronic band gaps are expected to emerge for such a scenario, as the sublattice symmetry of the graphene is slightly broken due to the in-plane strain field^10^,^12^,^14^,^15^. A large enough enhancement of the adhesion modulation should cause the graphene to snap into a globally commensurate CB-stacked phase (that is, graphene stretching uniformly to compensate for the lattice mismatch with hBN). The resulting heterostructure is expected to become a very high-mobility semiconductor with a sizable (∼50–200 meV) band gap^6^,^14^. Importantly, the strength of the adhesion modulation is controlled directly by the interlayer separation.

Here we demonstrate a path towards achieving control over this degree of freedom by demonstrating that pressure exerted by a scanning tunnelling microscope (STM) tip^16^,^17^,^18^,^19^,^20^ is capable of compressing or relaxing the interlayer separation locally between graphene and hBN. We also show that by modulating the interlayer separation we can control the degree of local commensurate stacking and the in-plane strain of graphene. This technique provides unprecedented control over the crystal structure of a two-dimensional (2D) vdW heterostructure.

## Results

### Lifting graphene with an STM tip

**Figure 1:**
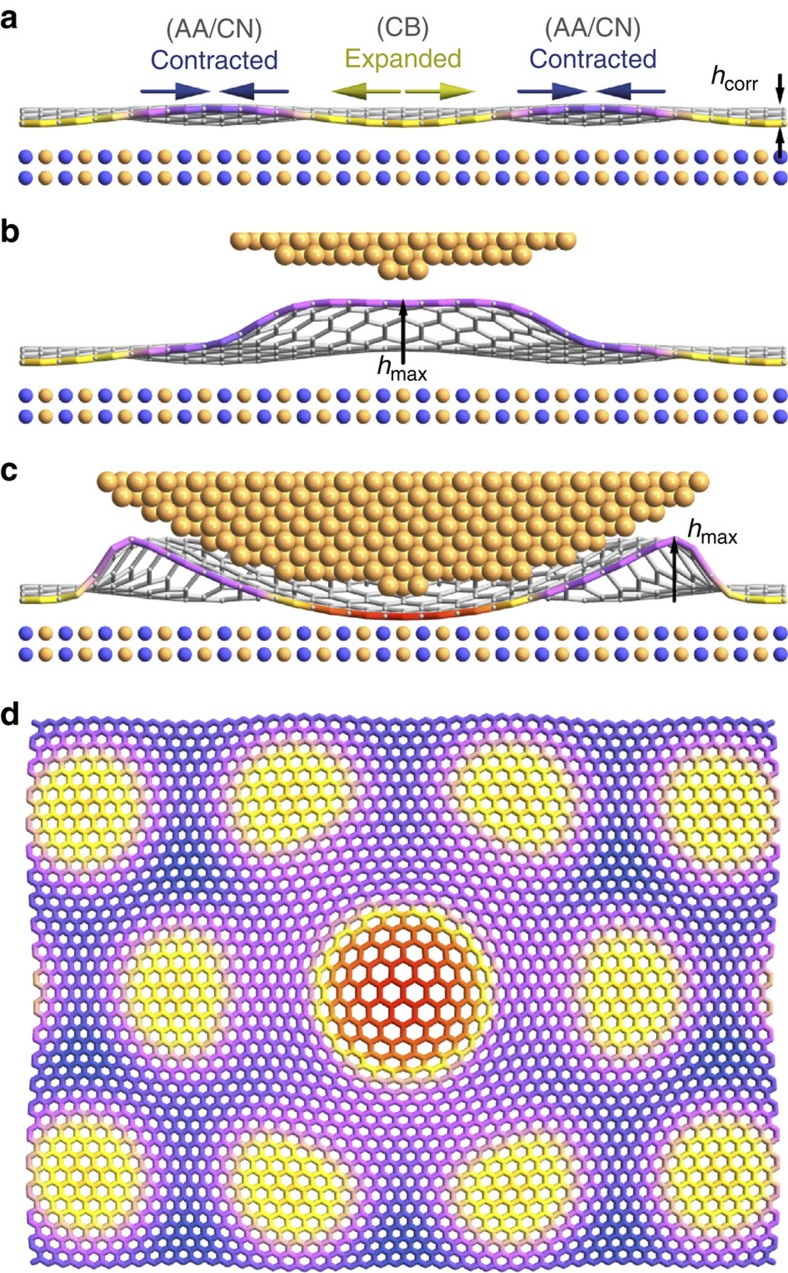
Schematic of graphene on hBN and the influence of an STM tip. (**a**) Schematic of an aligned graphene on hBN heterostructure. Due to the spatially modulated vdW adhesion potential, the graphene lattice periodically expands and contracts in-plane. An out-of-plane corrugation profile also develops, both matching the moiré. (**b**) In the presence of an STM tip, a vdW adhesion between the tip and graphene lifts the graphene off the surface of the hBN, modifying the strain field. (**c**) For an STM tip very close to the surface, the graphene is pushed closer to the hBN, enhancing the difference in the adhesion potential for different stacking configurations. The graphene lattice then expands to match the slightly longer lattice constant of the hBN. (**d**) Top view of (**c**), where the STM tip sits in the centre of a moiré period (that is, over a CB stacking configuration). The graphene lattice expands locally (red) to match the hBN lattice. Both the lattice constant and the spatial deformation have been scaled up for better visibility.

**Figure 2:**
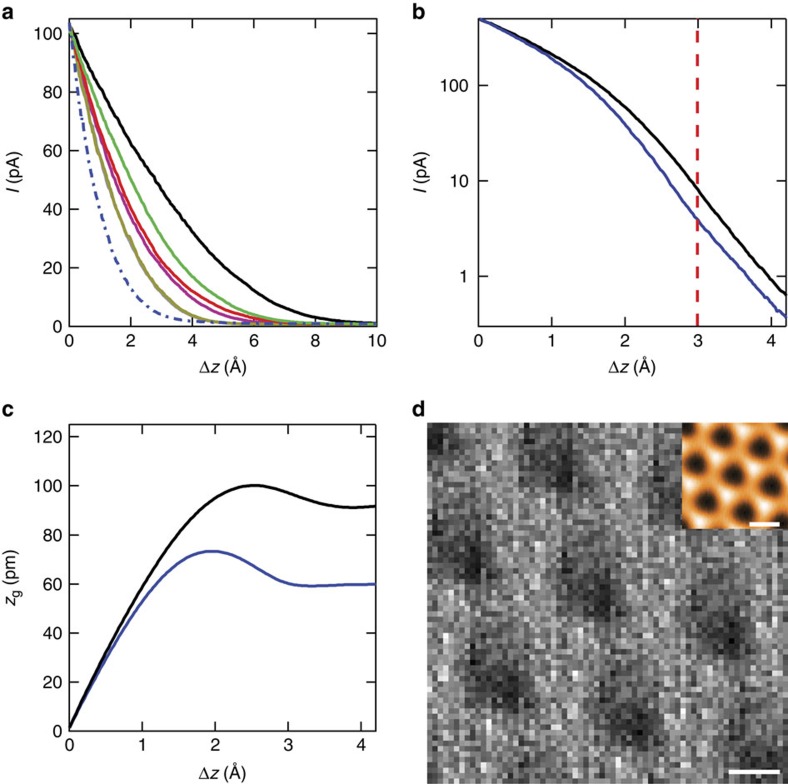
Tunnelling current as a function of tip-sample separation. (**a**) Measurement of the tunnel current *I* versus tip retraction distance Δ*z* for nearly-aligned graphene on hBN, starting with the tip in close proximity to the sample. The dot-dashed blue curve is taken on graphene on SiO_2_ for reference, and exhibits the anticipated exponential decay. The remaining curves, from gold to black, represent decreasing sample bias (that is, moving the tip closer to the surface), from 1 to 0.05 V. The decay is initially parabolic, and the crossover point to exponential decay grows to larger Δ*z* as the sample bias is lowered. (**b**) Similar decay measurement plotted on a log scale on a CB (blue) and CN/AA (black) region. The transition from parabolic to linear occurs at Δ*z* of about 2 Å. (**c**) Out-of-plane graphene movement relative to the hBN (*z*_g_) as a function of tip separation Δ*z*. As the tip is initially retracted, the graphene moves with it, lifting away from the hBN. At just over 2 Å, a maximum pulling distance is reached, and on further tip retraction the graphene slowly relaxes back towards the hBN. (**d**) Spatial map of the tunnelling current (dark is low and bright is high). The data are taken from the same set as in (**b**), at Δ*z*=3 Å. The inset displays the simultaneously acquired topography. The tunnelling current is smaller in the moiré centres than along the boundaries, suggesting a spatial modulation in the ability of the tip to pull the graphene off the hBN substrate. The maps have been spatially-averaged (see main text). Note that a similar pattern is exhibited at all Δ*z*, as the blue curve is always below the black in (**b**). The scale bar is 5 nm for the main panel and 10 nm for the inset.

We first present evidence of the out-of-plane movement of the graphene lattice produced by the tip, depicted schematically in Fig. 1b,c. We monitor the tunnel current *I* as a function of the relative tip-sample separation Δ*z*. The tunnelling current is expected to scale exponentially with Δ*z* as


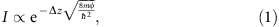


where *m* is the electron mass and *ϕ* is the tunnel barrier height. This exponential approximation holds well for graphene on SiO_2_, but fails for graphene on hBN (Fig. 2a), independent of relative rotation angle (Supplementary Note 2 and Supplementary Fig. 3). In the latter case, *I*(Δ*z*) becomes strongly dependent on the specific tunnelling parameters, with the tunnel current decay growing slower as the tip distance is brought closer to the surface. Furthermore, the decay is initially quadratic rather than exponential. Figure 2b shows a similar measurement with the tunnel current plotted on a logarithmic scale, further highlighting the initial regime of non-exponential decay. The departure from equation 1 implies that the graphene is moving with the STM tip as it retracts from the sample, owing to a vdW attraction between the graphene and the tip. The vdW adhesion is apparently stronger between the tip and graphene than between the graphene and hBN, as evidenced by a visible *I*(Δ*z*) hysteresis between tip approach and tip retraction (Supplementary Fig. 3). This allows the tip to modify the interlayer separation (while conversely, the graphene is more strongly adhered to the SiO_2_ substrate and is relatively immobile).

To account for the additional out-of-plane movement of the graphene sheet, we substitute Δ*z* in equation 1 with Δ*z*−*z*_g_(*z*), where *z*_g_(*z*) represents the movement of the graphene relative to the hBN substrate as a function of the tip position *z*. We plot the relative movement of the graphene in Fig. 2c, assuming an effective barrier height *ϕ*=4 eV, as extracted from measurements acquired at large tip-sample separations. The tip initially lifts the graphene away from the hBN as it retracts. After around 2 Å of retraction, the tip is no longer able to continue pulling the graphene, which then begins to slowly relax back towards the hBN substrate, as it is still under the influence of a vdW force from the tip^19^. It is important to note that the graphene is initially pushed towards the hBN by the tip, so the equilibrium separation lies somewhere at *z*_g_>0. The blue and black curves in Fig. 2b,c are taken in the centre and along the boundaries of the moiré, respectively, and exhibit a spatial variation in the maximum pulling amplitude of the tip. The variations can be further highlighted by plotting a spatial map of the tunnelling current at a fixed tip retraction distance Δ*z*, as in Fig. 2d. The spatial variation in the current matches the topographic moiré pattern, suggesting modulations in the magnitude of the out-of-plane graphene pulling by the tip due to the underlying spatial modulations in the adhesion potential between the graphene and the hBN.

### Modifying commensuration with interlayer spacing

**Figure 3:**
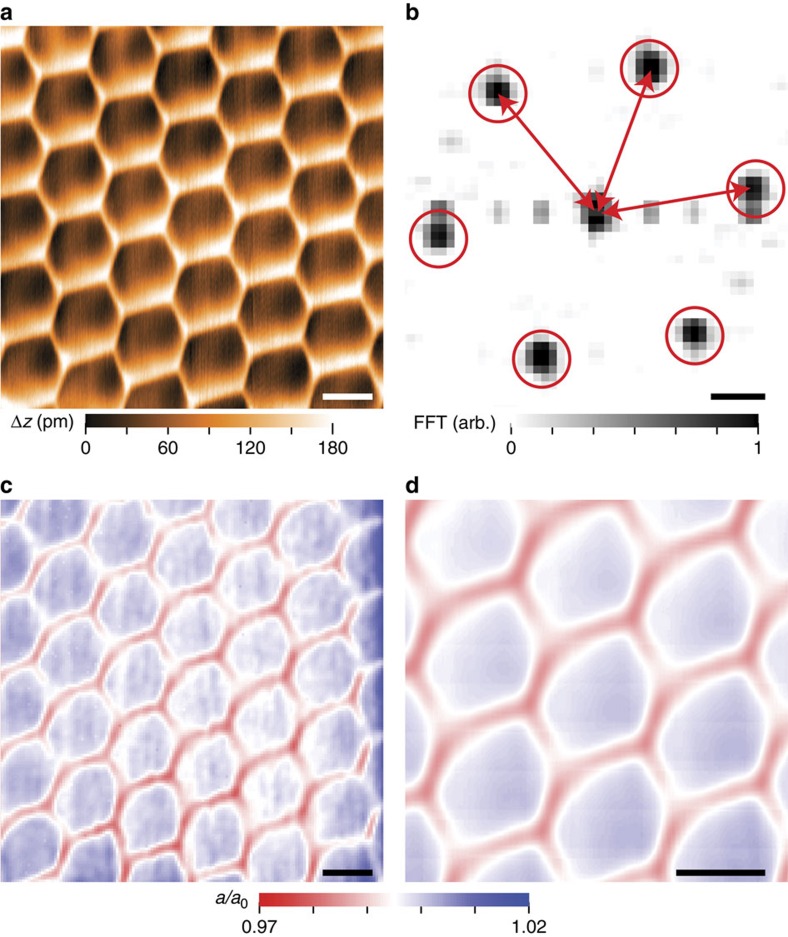
Method for generating strain maps. (**a**) Atomically-resolved topography of nearly-aligned graphene on hBN. The topography was acquired with a sample voltage of *V*_s_=0.3 V and a tunnelling current of *I*_t_=200 pA. (**b**) Fourier transform of a 4 × 4 nm^2^ region of (**a**), showing six resonances representing the hexagonal graphene lattice (red circles). The red arrows depict the measurement of the lattice constant in each direction. (**c**) Plot of the average length of the three lattice directions, as measured in (**b**) for each point in the topographic map. The points are normalized by the equilibrium graphene lattice constant *a*_0_. (**d**) Spatially-averaged strain map, generated by averaging (**c**) over a few moiré unit cells. The scale bars are 10 nm for (**a**,**c**,**d**) and 10 nm^−1^ for (**b**).

The relative adhesion potentials between the CB, CN (carbon–nitrogen) and AA (hexagons atop one another) stacking configurations depend on the interlayer separation between the two materials (Supplementary Note 4 and Supplementary Fig. 6). To understand how the in-plane strains in the graphene lattice depend on the interlayer separation, and to show how they can be controlled through tip pressure, we have acquired atomically-resolved topographic maps of nearly-aligned graphene on hBN heterostructures (Fig. 3a) with varying tunnel resistance (which controls tip-sample separation and therefore the interlayer separation). All measurements were performed in ultra-high vacuum at a temperature of 4.5 K. From a topographic map, we take small (4 × 4 nm^2^) areas, perform a Fourier transform (Fig. 3b), and extract the average length *a* of the three resonances due to the hexagonal graphene lattice. We then create a map of the average graphene lattice constant normalized by the equilibrium length (*a*/*a*_0_, with *a*_0_=2.46 Å) as a function of position (Fig. 3c). Finally, to enhance the clarity of these strain images we average each point in the moiré unit cell with all other equivalent sites in the strain image (Fig. 3d).

**Figure 4:**
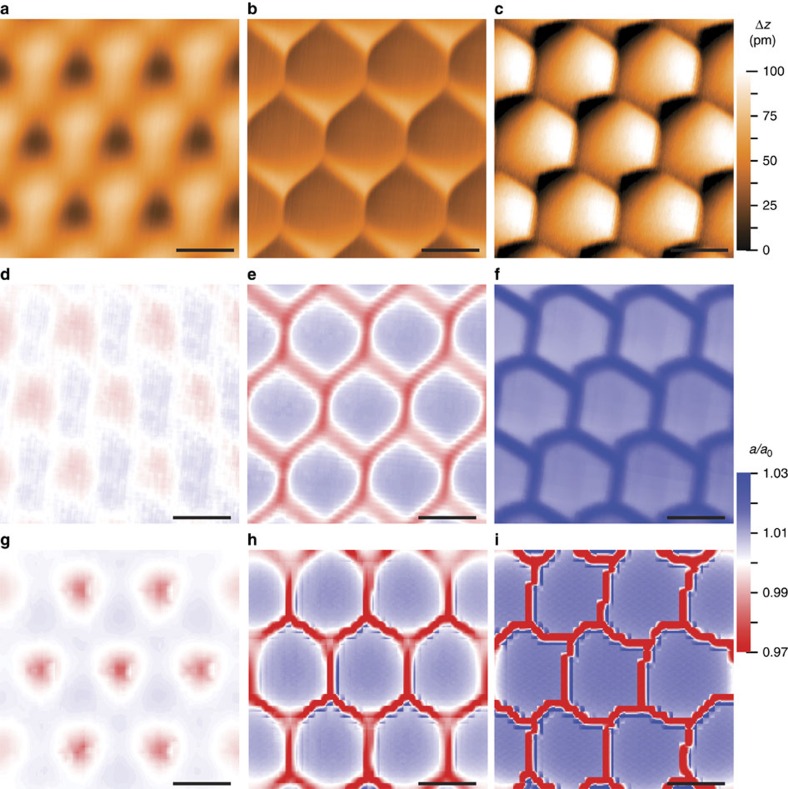
Topography and strain maps in different interaction regimes. (**a**–**c**) Spatially-averaged topography maps acquired over the same region of a nearly-aligned sample, with the tip moving progressively closer to the sample. The appearance of the stacking boundaries becomes sharper as the tip moves closer, and becomes hysteretic and asymmetric in (**c**). (**d**–**f**) Experimental spatially-averaged strain maps generated from the topographic maps of (**a**–**c**). In (**d**) the graphene lattice is compressed in the moiré centres and expanded along the boundaries. The opposite behaviour is observed in (**e**). In (**f**), the graphene lattice constant for the entire map is expanded, as the system is in a strongly interacting, hysteretic regime. (**g**–**i**) Simulated strain maps, showing excellent agreement with the experimental results. The disagreement in the dynamical strain at the stacking boundaries between (**f**) and (**i**) is attributed to the absence of out-of-plane buckling in the simulation. The tunnelling parameters are (**a**) *V*_s_=0.5 V and *I*_t_=50 pA, (**b**) *V*_s_=0.5 V and *I*_t_=900 pA and (**c**) *V*_s_=0.05 V and *I*_t_=100 pA. All scale bars are 10 nm.

Figure 4a–c shows spatially-averaged STM topography images taken over the same area of a nearly-aligned graphene on hBN sample with decreasing tip-sample separation. The hexagonal stacking boundaries in the measured moiré pattern grow sharper as the tip moves closer to the surface, exerting an increasing pressure. Below a critical tip separation, the stacking boundaries appear atomically and sub-atomically sharp, and a hysteresis eventually develops in their positions between the forward and backward scan directions (Fig. 4c; Supplementary Fig. 1). This observation clearly points to a strong influence of the tip on the graphene lattice. If the sample were unperturbed by the tip, the appearance of the topography, and in particular the measured thickness of the stacking boundaries would correspond to the equilibrium sample configuration, and should not depend on the tip pressure except for a local density of states component which can be eliminated (Supplementary Note 1). The graphene lattice strain maps for the different characteristic profiles of the moiré topography are shown in Fig. 4d–f. Like the topography, these are not equilibrium strain fields but rather local strains under the tip that dynamically evolve during the scan in response to the moving tip interaction. We identify three typical and qualitatively different spatial patterns in this dynamical strain. Stacking boundaries can appear thick, but are expanded relative to the CB regions (large tip-sample separations, Fig. 4d). This is opposite to the equilibrium expectation. Alternatively, boundaries can appear thin, and are compressed relative to CB regions (intermediate tip separations, Fig. 4e). Finally, boundaries can exhibit hysteretic behaviour and broken three-fold symmetry, and the entire graphene lattice is expanded relative to equilibrium (smallest tip separations, Fig. 4f). The response of the sample to the tip is so strong that, within the limits of our STM measurements, it is never possible to measure the equilibrium configuration of the heterostructure (that is, even at very large tip-sample separations, the graphene is still lifted off the hBN). The apparently sharp boundaries in Fig. 4c in particular, also observed in our previous work^21^, are therefore not an equilibrium configuration.

Interestingly, we observe qualitatively similar behaviour in slightly misaligned samples as well. Specifically, we observe the three different strain profiles as a function of tip-sample separation in all moiré areas studied with periods varying from 14 nm (essentially perfect alignment) down to about 6 nm (below which the behaviour may persist, but our analysis is no longer sensitive as the size of our Fourier transform window becomes comparable to the entire moiré unit cell). As an example, Supplementary Fig. 2 shows strain maps for an 8 nm moiré period. This observation is in stark contrast to the results of ref. 22, the reasons for which will be discussed in our model below and in Supplementary Note 6.

### Theoretical analysis

We have simulated the dynamical strain of the graphene lattice under a scanning tip using a simple adhesion model between graphene and hBN (see Methods and Supplementary Note 4 for full details, as well as Supplementary Movies 1–4 for animations). In our model, the graphene sticks to a parabolic tip, and can thus be locally compressed against or separated away from the hBN substrate. Figure 4g–i shows the strain maps obtained for decreasing tip-sample separations, which exhibit excellent agreement overall, both qualitatively and quantitatively with their experimental counterparts. The three characteristic spatial patterns arise naturally when the effective interaction between the tip and the equilibrium stacking boundaries changes with *z* from attractive, to repulsive, and to strongly repulsive. In the attractive regime, the graphene under the tip is lifted off the hBN surface, lowering the adhesion potential. The stacking boundaries are then attracted to the scanning tip, and as a result the graphene lattice appears to be expanded along the stacking boundaries (Fig. 4g). In the repulsive regime, the tip is pushing down on the sample, increasing the adhesion energy modulation. The CB-stacked regions then become expanded under the tip, up to the maximum static value *a*/*a*_0_=1+*δ* (local commensurate stacking) at high pressure, and the stacking boundaries are pushed away (Fig. 1d for a schematic of the graphene lattice strain when the tip sits above the CB centre of the moiré). As the tip scans the sample, the commensurate area underneath (red in the schematic) moves with it, and the stacking boundaries are likewise pushed along (Fig. 4h). If the tip pressure is strong enough, the stacking boundaries are pushed until, eventually, they irreversibly snap back under the tip (Fig. 4i). This abrupt snapping results in the observed hysteretic behaviour with tip scan direction, and a breaking of the characteristic three-fold symmetry of the moiré pattern (note that the expanded hysteretic boundaries that develop in this regime may be explained by sudden out-of-plane delamination of graphene in front of the tip, a possibility not included in our model, see Supplementary Note 4).

The notable success of our simulations in reproducing the experimental dynamical strain maps allows us to confidently remove the tip from the simulations, to understand the equilibrium configuration of the graphene lattice. We find that the observed phenomenology is consistent with intrinsic adhesion potential differences^23^,^24^ of *V*_AA_−*V*_CB_=16 meV per graphene unit cell, similar to the values from *ab-initio* calculations^11^. Importantly, our results are not consistent with an adhesion potential difference of zero (nor an infinitely stiff graphene lattice). The corresponding strain of the graphene at equilibrium (without a tip) is rather weak, and varies almost sinusoidally between ±0.3% (Supplementary Fig. 10). This is in stark contrast to the dynamical strain maps, which may appear much sharper spatially and in excess of ±1%. These dynamical strain effects are important to consider in all scanning probe measurements of graphene on hBN (refs 9, 22) (Supplementary Note 6).

## Discussion

We have demonstrated unprecedented control of the atomic structure of graphene by locally modifying the interaction strength with an hBN substrate through pressure applied with an STM tip. This allowed us in particular to induce and directly image tunable in-plane strains and local commensurate stacking. While a globally commensurate graphene on hBN structure is expected to exhibit an electronic band gap, we do not observe any signatures of a gap in our tunnelling spectroscopy measurements of the local density of states (Supplementary Note 3) for any applied tip pressure. When the tip is far from the sample, such that it remains incommensurate, the tip likely screens the many-body interactions responsible for the development of the band gap typically observed in transport experiments^14^,^25^,^26^. When the graphene is commensurate with the hBN, the gap is expected to be of order 50 meV even before the consideration of potential many-body enhancement^6^. Therefore, it may seem surprising that we also do not observe a band gap in tunnelling spectroscopy even in the case where the tip is very close to the sample, such that the graphene is commensurate with the hBN underneath the tip. However, the lack of observed band gap is a consequence of the local nature of the applied pressure in our experimental setup. A gap of magnitude Δ corresponds to the localization of states of typical wavelength *λ*_Δ_=*hv*_F_ /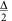. For the anticipated band gap Δ≈50 meV, states must be localized on length scales of order 100 nm. In our work, our model predicts that the area of the graphene forced into a commensurate state with the hBN is confined to approximately one moiré period, of order 10 nm (Fig. 1d). Thus, the lack of a band gap in tunnelling spectroscopy is to be expected because the commensurate area is considerably smaller than the requisite localization area (Supplementary Notes 3 and 5 for further details about the tunnelling spectroscopy measurements and their theoretical modelling).

This suggests a natural extension of our work, where a graphene sheet is forced into a commensurate state with hBN over the entire sample area. Fortunately, the technique of applying pressure to a vdW heterostructure is very easily generalizable to the scale of the entire device using hydrostatic or diamond anvil pressure cells. In graphene on hBN in particular, we anticipate a globally commensurate state to emerge under a hydrostatic pressure of roughly 150 MPa (Supplementary Note 4 and Supplementary Fig. 8), characterized by the absence of a moiré pattern and a large band gap due to globally broken sublattice symmetry in the graphene. More generally, global control of the interlayer separation through pressure in other vdW heterostructures should enable exciting new experimental designs, and result in the emergence of many novel electronic device properties.

## Methods

### Sample preparation and measurement details

Chemical vapour deposition grown graphene was transfered onto mechanically exfoliated hexagonal boron nitride resting on a Si/SiO_2_ substrate. The devices were annealed at 350 °C in a mixture of argon and hydrogen, then at 300 °C in air. Similar results to those reported here were observed in preliminary work with exfoliated graphene flakes as well.

All the STM measurements were performed in ultra-high vacuum at a temperature of 4.5 K using a tungsten tip. The tunnelling resistance was varied over five orders of magnitude by controlling the sample bias and tunnelling current. We note that tip geometries are somewhat random between different tips, and between different tip shaping procedures on the same tip. Because the nature of the tip ending is also important for determining the interaction strength with the substrate, comparing tunnelling resistances between different measurements is not itself a sufficient metric for determining the amount of compression or relaxation of the graphene relative to the hBN.

### Tip preparation

Tungsten tips were prepared by electrochemical etching, and further shaped *in situ* when necessary by applying electrical pulses of 5–10 V on the Au contacts far from the graphene sample. The lattice deformation effects detailed here have been observed with every tip (tens of tips measured in total) and over tens of pulse cycles per tip. We note that qualitatively similar moiré scale lattice deformations have been observed in graphene on Ir(111) with AFM using a tip intentionally terminated with a carbon monoxide molecule^27^. While we cannot rule out that a deformable tip could have some influence on our results, we are confident that the primary source of the effects we present can be explained by our proposed model for a number of reasons. First, because we do not intentionally terminate our tips with a deformable molecule, it is very unlikely that we would observe similar results across all of our tips and pulse cycles if such a deformable tip ending were being randomly picked up every time. Second, the deformable tip ending would have to be metallic to be relevant for our tunnelling measurements. While our samples may have water, hydrogen or other small molecule adsorbates, they should certainly be free of metallic contaminants to unintentionally attach to the end of every tip. Further, we observe our reported behaviour even with brand new tips, which are landed directly onto the graphene. Third, we observe sub-atomically sharp discontinuities in the topography only on the moiré length scale (in contrast to previous reports showing such behaviour on the atomic scale using a cobalt atom dragged across the surface of the sample^28^). No similar model can easily explain our observation of smooth atoms except at moiré boundaries in the hysteretic regime, which would require a much longer deformation length scale and a strong preference for irreversible topographic discontinuities only at special sites on the moiré. This suggests the discontinuities instead arise from lattice deformations in the graphene at moiré boundaries as we argue in our model. Finally, we observe a saturation of the graphene lattice constant expansion at just under 2% in the hysteretic regime (excluding the boundaries, which exhibit irreversible discontinuities), consistent with a commensurate structural transition (as this is roughly the lattice mismatch between graphene and hBN). We have never observed significantly larger lattice deformations. We would not anticipate such a bound if this effect were due to a deformable tip, providing further compelling evidence that the apparent lattice deformations we observe are primarily due to a modification of the graphene lattice itself, as proposed in our model.

### Theoretical model

An overview of our theoretical model is as follows (see Supplementary Note 4 for full details). The STM tip is approximated by a paraboloid of radius *R* around its apex, hovering at height *h*_0_ relative to a relaxed reference plane (taken as the graphene position at the CB-stacked regions—recall that graphene is slightly corrugated due to non-uniform adhesion to hBN). We assume that the vertical graphene displacement conforms to the tip profile as long as it does not exceed a certain height, *h*_max_, see Fig. 1c. Otherwise graphene takes on the equilibrium vertical displacements at each stacking. We assume a certain in-plane distortion ***u***(***r***) of the sample, relative to the relaxed moiré pattern, which we want to determine. We construct a smooth interpolation of the *ab-initio* adhesion potentials *V*_S_(*z*) between different graphene/hBN stackings, where *z* is the separation between the two crystals. Using the interpolated potential, we evaluate the total adhesion energy per unit area for a given field ***u***(***r***). At each ***r***, the value of *z* is constrained by the tip profile, as described above. To this adhesion energy, we add the corresponding elastic energy associated to ***u***(***r***). We discretize ***r***, and express the total energy as a function of the finite set of ***u*** on the discrete mesh. We minimize the total energy, using conjugate gradient methods, and find the deformation ***u***(***r***) at equilibrium. We then obtain the dynamical strain as measured by the tip by performing this sample relaxation as the tip moves across the sample at a constant height *h*_0_. The model has no unconstrained free parameters, as all can be roughly estimated experimentally.

### Data availability

The data that support the findings of this study are available from the corresponding author on request.

## Additional information

**How to cite this article:** Yankowitz, M. *et al*. Pressure-induced commensurate stacking of graphene on boron nitride. *Nat. Commun.* **7,** 13168 doi: 10.1038/ncomms13168 (2016).

## Supplementary Material

Supplementary Information Supplementary Figures 1-13, Supplementary Table 1, Supplementary Notes 1-6 and Supplementary References

Supplementary Movie 1 Movement of the scanning probe tip across the moiré at 0.8 Angstrom above the equilibrium graphene position.

Supplementary Movie 2 Movement of the scanning probe tip across the moiré at 0.2 Angstrom above the equilibrium graphene position.

Supplementary Movie 3 Movement of the scanning probe tip across the moiré at the equilibrium graphene position.

Supplementary Movie 4 Movement of the scanning probe tip across the moiré at -0.2 Angstrom above the equilibrium graphene position.
