# Supplementary material for: Pressure-induced commensurate stacking of graphene on boron nitride
Source: Nat Commun. 2016 Oct 20;7:13168. doi: 10.1038/ncomms13168 (PMC5462001; doi:10.1038/ncomms13168)
Supplement: Supplementary Information — Supplementary Figures 1-13, Supplementary Table 1, Supplementary Notes 1-6 and Supplementary References [file ncomms13168-s1.pdf]

SUPPLEMENTARY FIGURES

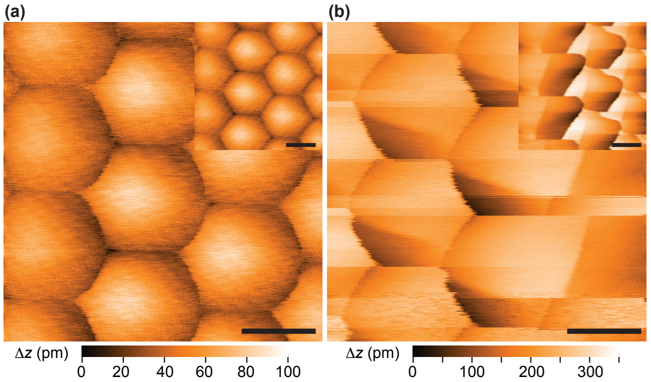

Supplementary Figure 1. Topography in the hysteric regime. The tip gets closer to the sample from (a) to (b). The tip scans from left to right in both. The respective insets show the opposite scanning direction. The walls are pushed in the scanning direction of the tip. The walls are (a) atomically and (b) sub-atomically sharp, indicating a non-equilibrium measurement of the graphene, and are pushed further as the tip is in a more invasive regime. The tunneling parameters are (a) $V_s = 0.3$ V and $I_t = 150$ pA and (b) $V_s = 0.025$ V and $I_t = 150$ pA. All scale bars are 10 nm.

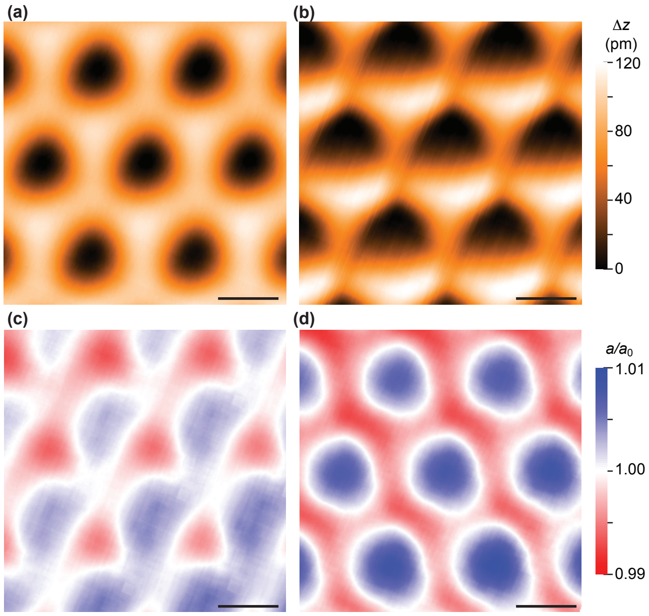

Supplementary Figure 2. Topography and strain maps in different interaction regimes. (a)-(b) Spatially-averaged topography maps acquired over the same region of a slightly-misaligned sample (8 nm moiré), with the tip moving progressively closer to the sample. (c)-(d) Spatially-averaged strain maps generated from the topographic maps of (a)-(b). In (c) the graphene lattice is compressed in the moiré centers and expanded along the boundaries. The opposite behavior is observed in (d). These behaviors are consistent with the nearly-aligned samples. The tunneling parameters are (a) V_s = 0.95 V and I_t = 100 pA and (b) V_s = -0.15 V and I_t = 100 pA. All scale bars are 5 nm.

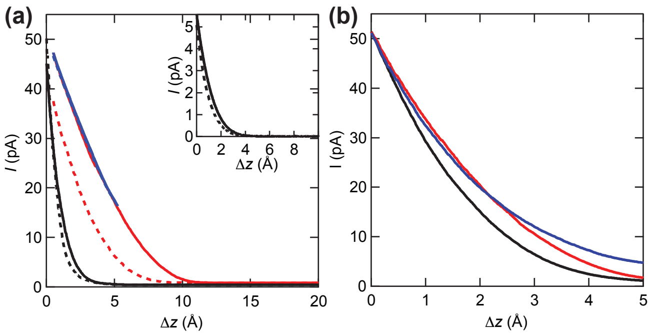

Supplementary Figure 3. Decay of the tunneling current. (a) Tunneling current versus Δz for both the tip retracting (solid) and approaching (dashed) the sample. The black curves are acquired on graphene on SiO_2, exhibiting roughly exponential decay and virtually no hysteresis. The red curve is acquired on graphene on hBN, showing significant departure from exponential decay and large hysteresis. The blue curve is acquired similarly, but with only a small retraction such that the tunneling current is always finite. In this case, no hysteresis is observed. (Inset) Graphene on hBN with a very large initial tip-sample separation. In this case, the decay is nearly exponential and non-hysteric. (b) Decay curves for misaligned graphene on hBN for decreasing initial tip-sample separations (black to red to blue). Similar behavior to the nearly-aligned case is observed, marked by significant departure from exponential decay.

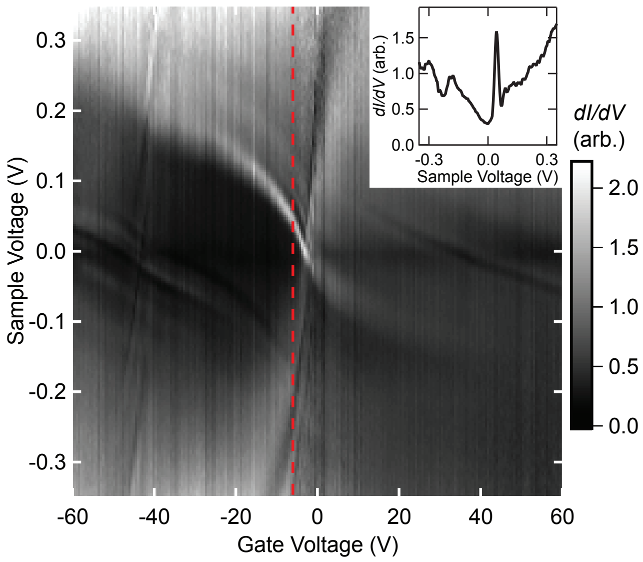

Supplementary Figure 4. Gate map of nearly-aligned graphene on hBN acquired in a strongly invasive regime. The main Dirac point is prominently visible, though there is no obvious associated band gap. Replica Dirac point features are also observed in the conduction and valence bands. (Inset) Cut of the dI/dV taken at V_g = -5 V (the charge neutrality point). The density of states at the Dirac point remains finite, implying the lack of a band gap.

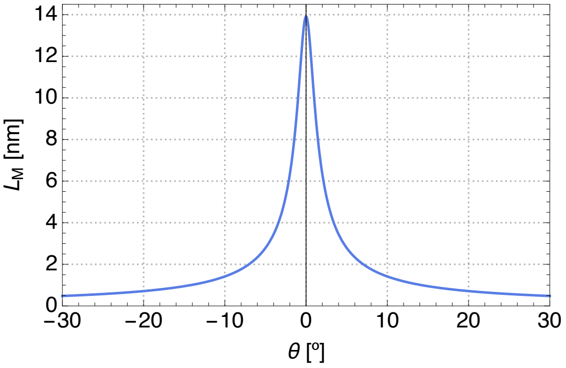

Supplementary Figure 5. The moiré period $L_{\mathrm{M}} = |\hat{A}_{\mathrm{i}}|$ as a function of twist angle θ, for mismatch $\delta = 1.8\%$.

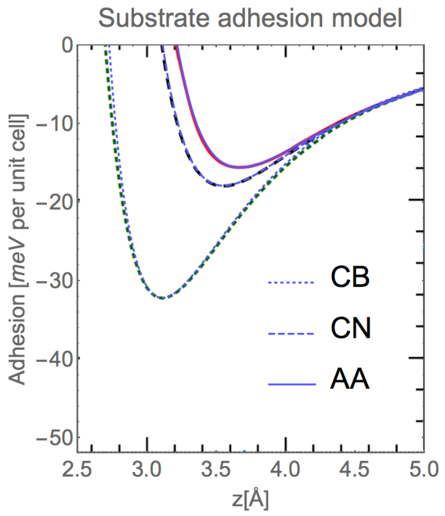

Supplementary Figure 6. Theoretical adhesion energy versus stacking. Fit (in blue) to microscopic results (Ref. [1], in red, black and green, reproduced here with permission) for the adhesion energy $V_\alpha(z)$ at different stackings $\alpha = AA, CN, CB$.

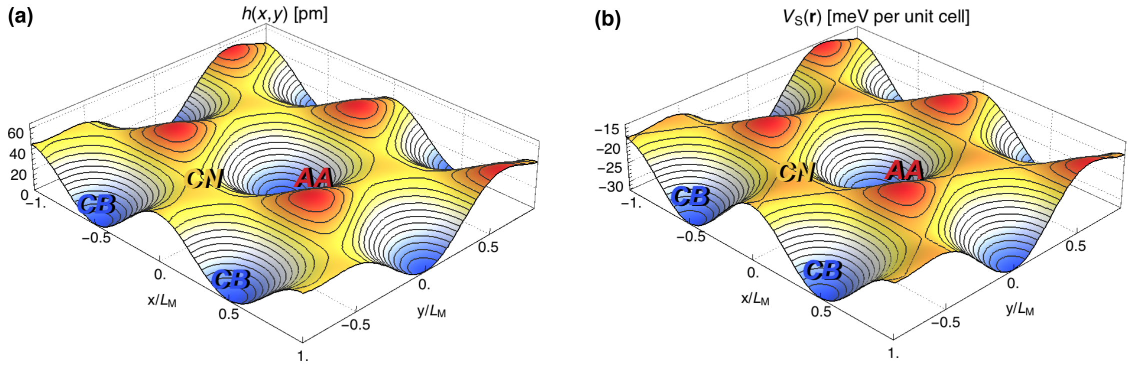

Supplementary Figure 7. Height and potential landscape on the moiré cell. (a) Substrate-induced corrugation $h(\mathbf{r})$ of the graphene sample at equilibrium, relative to the $z = d_{\mathrm{CB}}$ plane. (b) Adhesion energy to the substrate per graphene unit cell $V_{\mathrm{S}}(\mathbf{r})$.

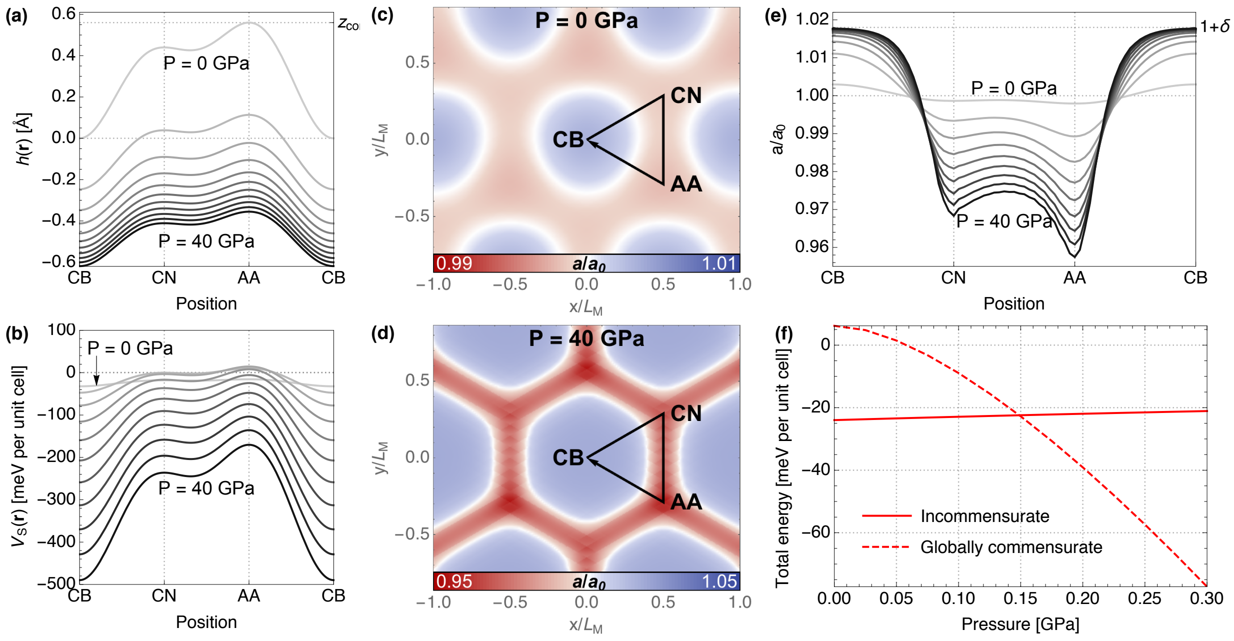

Supplementary Figure 8. Graphene strain and energy versus hydrostatic pressure. Pressure dependence of (a) equilibrium corrugation $h(\mathbf{r})$ and (b) adhesion energy per unit cell, $V_S(\mathbf{r}) + V_P(d_{CB} + h(\mathbf{r}))$. Normalized average lattice constant $a/a_0 = \frac{1}{2}\mathrm{Tr}\hat{u} + 1$ at equilibrium for (c) zero hydrostatic pressure $P = 0$, and (d) high pressure $P = 40$ GPa. (e) Cut of local normalized lattice constant a/a_0 along the white path marked in (c,d), as pressure is increased. In all cases, the total area of the sample is forced to remain unchanged, thus precluding a transition to a globally commensurate phase with uniform $a/a_0 = 1+\delta \approx 1.018$. (f) Total energy (elastic plus adhesion) per graphene unit cell at equilibrium versus pressure (solid line). The energy is measured relative to that of graphene in vacuum ($U = 0$). The dashed line corresponds to the energy of the globally commensurate sample, with uniform strain δ and $h(\mathbf{r}) = 0$. Said configuration is preferred over the incommensurate moiré strain superlattice above a critical pressure $P_c = 0.15$ GPa.

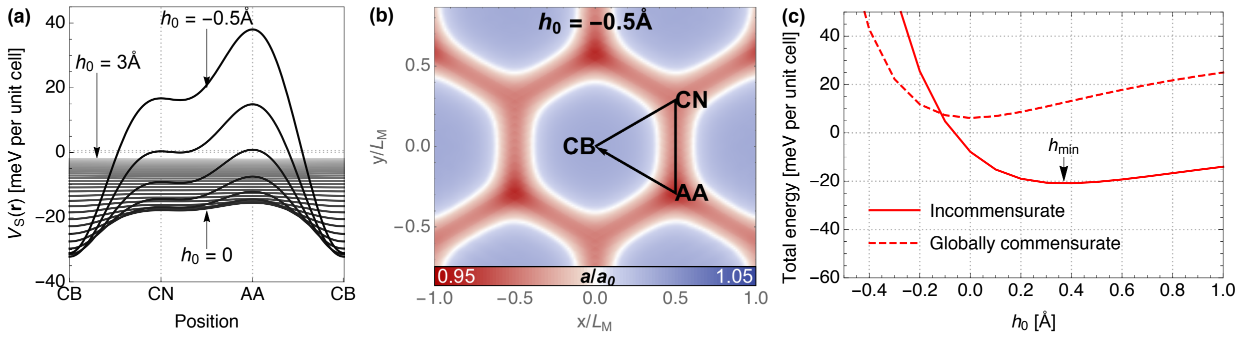

Supplementary Figure 9. Graphene strain and energy under a flat metal plate. (a) Adhesion energy per graphene unit cell, assuming a pinning of the sample/substrate distance to a constant $z = d_{\rm CB} + h_0$ (e.g. by contacting the sample with a flat metallic plate at z). Note that for negative h_0 adhesion energy differences are enhanced (analogous to high hydrostatic pressure), while a positive h_0 suppresses adhesion differences. (b) Equilibrium normalized lattice constant a/a_0 profile in the incommensurate phase with $h_0 = -0.5$Å(similar to the $P = 40$ GPa case of Supplementary Figure 8(d)). (c) Total energy relative to graphene in vacuum ($h_0 = \infty$). The globally commensurate phase (dashed) becomes energetically favorable for $h_0 < -0.12$Å.

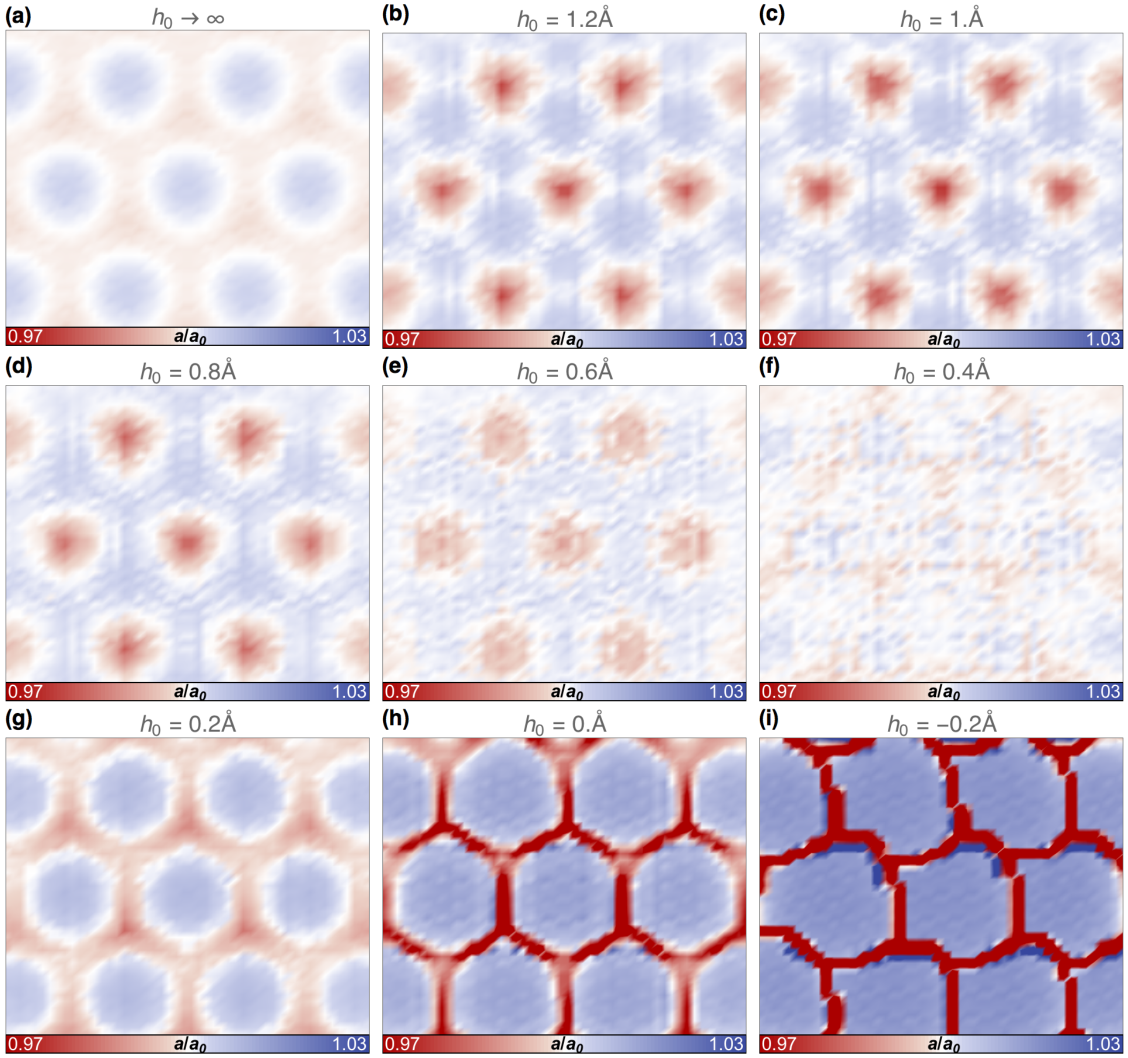

Supplementary Figure 10. Dynamical strain as measured by a tip scanning at different tip-sample distances h_0. The tip has a radius $R_{\text{tip}} = 200$ nm, and the maximum lift height is $h_{\max} = 1.5$ Å. Panel (a) shows the equilibrium (static) strain without the tip ($h_0 > h_{\max}$). Panels (b)-(e) show the attractive regime $h_{\min} < h_0 < h_{\max}$, where $h_{\min} = 0.37$Å. Panel (f) is at a crossover $h_0 \approx h_{\min}$. Panels (g) and (h) show the non-hysteretic repulsive regime $0 \lesssim h_0 < h_{\min}$, and (i) shows the hysteretic repulsive regime $h_0 \lesssim 0$. The small hexagonal mesh in each is an artifact of the mesh size of the simulation.

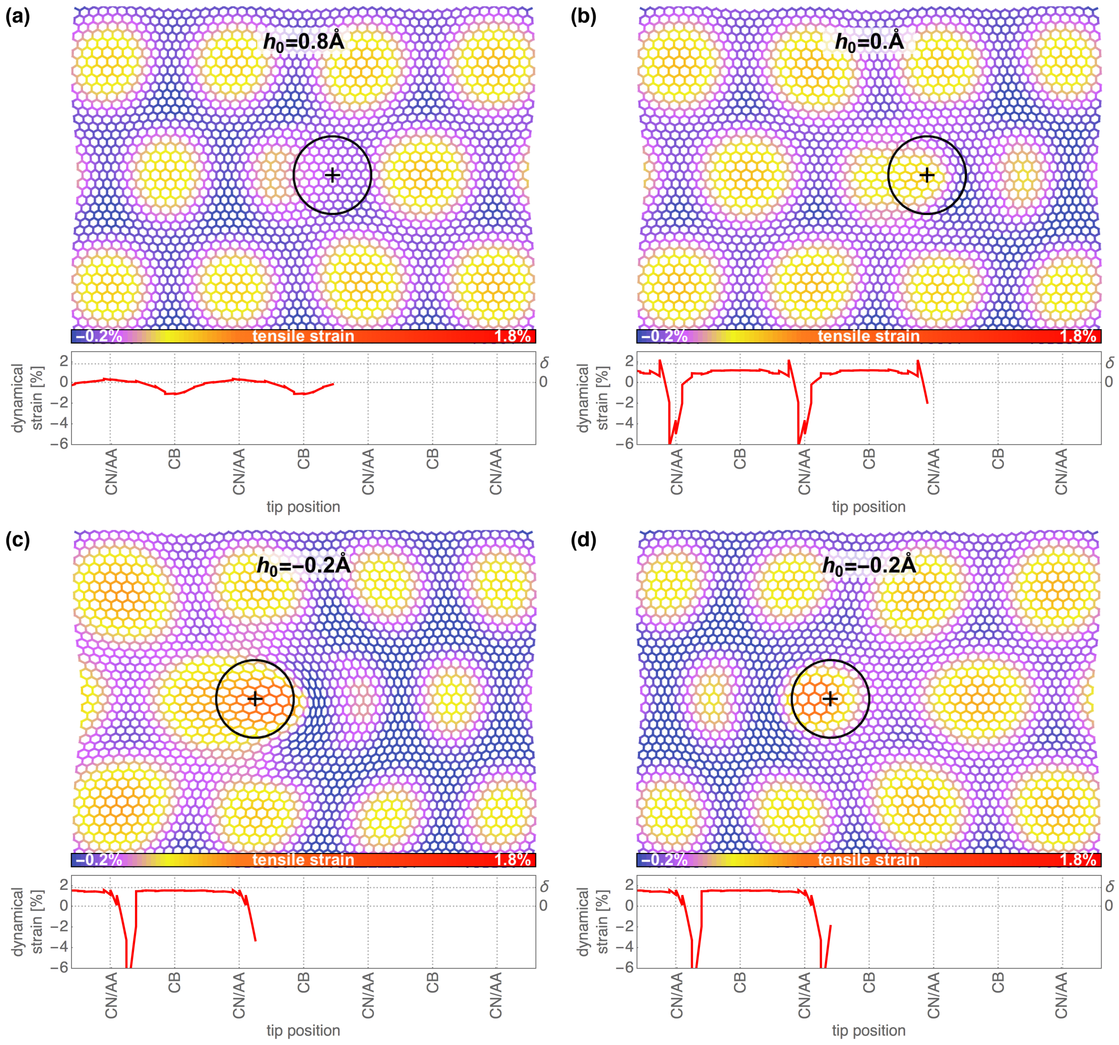

Supplementary Figure 11. Snapshots of Supplementary Movies 1-4. The snapshots depict the time evolution of both the instantaneous strain (top view) and the dynamical strain (bottom view) under a tip, scanning from left to right across the middle of the sample. We show the attractive (a), the repulsive non-hysteretic (b) and the repulsive hysteretic regimes (c) and (d). The tip position in the plane is shown in black, with its distance to the sample shown by the h_0 label. Both the lattice constant and the spatial deformation have been scaled up for better visibility in the top views (the color scale and bottom views represent the actual, unscaled strain).

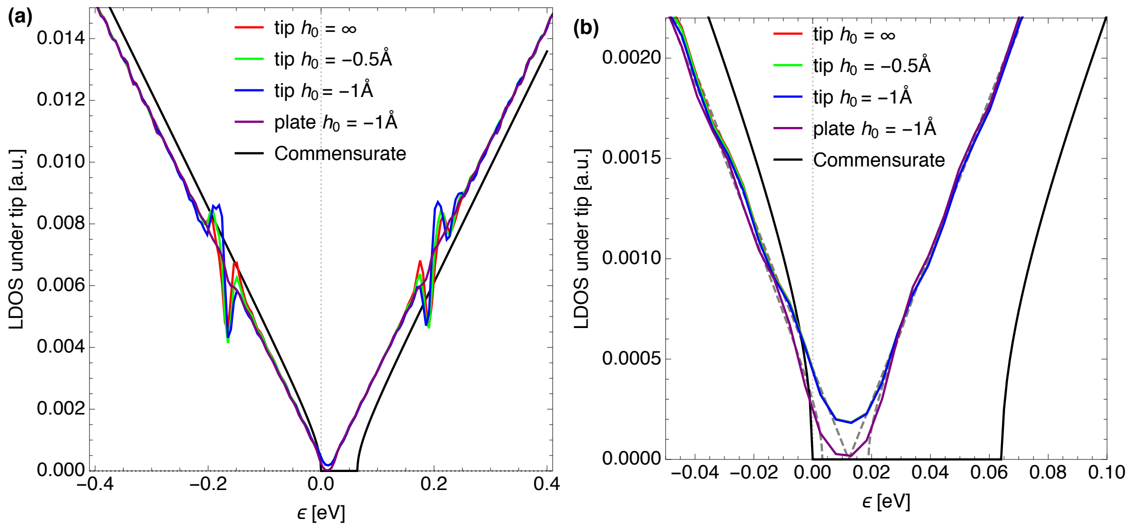

Supplementary Figure 12. Local density of states (LDOS) under the tip (at the center of a CB stacking region) for different tip heights h_0, including the effect of pressure-induced deformations. In purple, the LDOS for a flat plate at height $h_0 = -1$Å. The dashed line in the zoom-in (panel b), correspond to LDOS fits to a gapped Dirac spectrum, that show a negligible gap under tip pressure (because of the finite area of the deformed region), and the finite gap under the plate. The black line corresponds to the LDOS for the globally commensurate phase, with a ~ 64 meV gap without many-body corrections.

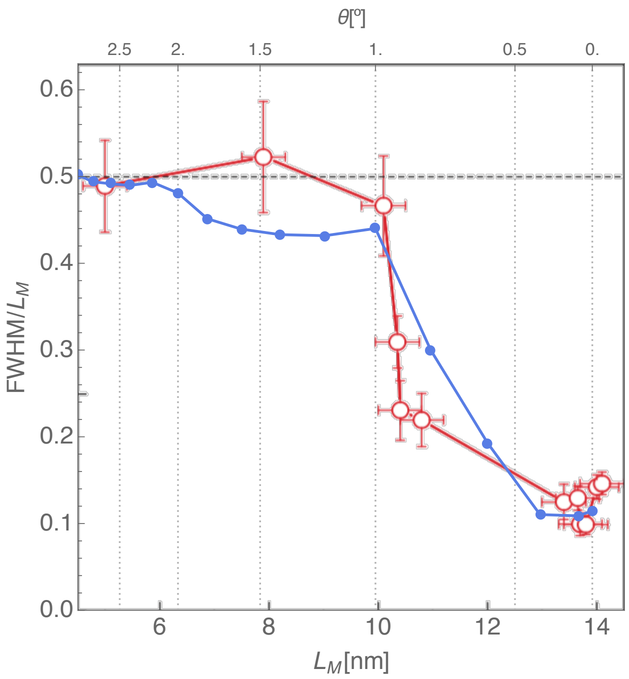

Supplementary Figure 13. Apparent lattice strain versus moiré period. In blue, full-width at half-maximum (FWHM) of the apparent strain, Supplementary Equation 28, across the center of a stacking boundary, as a function of twist angle θ. In red, FWHM reported in Ref. [2], reproduced here with permission.

SUPPLEMENTARY TABLES

$d_{\mathrm{AA}}[\text{Å}]$	$V_{\mathrm{AA}}\left[\frac{\mathrm{meV}}{\mathrm{cell}}\right]$	$d_{\mathrm{CB}}[\text{Å}]$	$V_{\mathrm{CB}}\left[\frac{\mathrm{meV}}{\mathrm{cell}}\right]$	$d_{\mathrm{CN}}[\text{Å}]$	$V_{\mathrm{CN}}\left[\frac{\mathrm{meV}}{\mathrm{cell}}\right]$
3.67	−15.6	3.11	−32.2	3.55	−17.9

Supplementary Table 1. Fit parameters for data in Supplementary Figure 6 using model of Supplementary Equation 9.

SUPPLEMENTARY NOTE 1 - CONSTANT TUNNELING CURRENT STRAIN MAPS

The interaction strength between the STM tip and the graphene/hBN heterostructure can be tuned experimentally by varying the tunneling resistance. The tunneling current can be expressed as

$$I \approx \frac{4\pi e}{\hbar} e^{-\Delta z\sqrt{\frac{8m\phi}{\hbar^2}}} \rho_{\mathrm{t}} \int_{-eV}^{0} \rho_{\mathrm{s}}(\varepsilon) d\varepsilon, \qquad \text{(Supplementary Equation 1)}$$

where Δz is the relative separation between the tip and sample, ϕ is the tunneling barrier height (essentially the average work function of the tip and sample), $\rho_{\mathrm{s(t)}}(\varepsilon)$ is the density of states of the sample (tip), m is the bare electron mass and e is the charge of the electron. Topography measurements are performed using a feedback loop to maintain a constant tunneling current I. Through a simple Ohm's law relationship, $R = V/I$, it is easy to see that lowering the tunneling resistance requires either lowering the bias voltage V or raising the tunneling current I. In the latter case, Supplementary Equation 1 implies that this requires lowering the tip-sample separation z, thus increasing the vdW interaction strength between the tip and sample.

Controlling the vdW interaction strength through the sample bias V is more complicated however, as the local density of states of the sample depends on energy. Therefore, in principle, large differences in the LDOS as a function of energy could dominate Supplementary Equation 1, perhaps artificially changing the appearance of the topography (rather than the dynamic interaction proposed in the main text). Although this is unlikely to also explain the differences in the strain maps, we conclusively eliminate this effect by acquiring topography maps at a fixed sample bias and varying tunneling current. Figs. 4(a) and (b) of the main text were acquired at the same sample bias with varying tunneling current, to ensure that the switch from the attractive to repulsive regimes could be achieved without any LDOS variation. Similarly, the repulsive regime can be driven hysteretic by varying only the tunneling current as well.

SUPPLEMENTARY NOTE 2 - DECAY OF TUNNELING CURRENT

In Fig. 2(a) of the main text, we track the decay of the tunneling current upon tip retraction for nearly-aligned graphene on hBN. In Supplementary Figure 3(a) we also track

the tunneling current upon reapproaching the tip. The solid black and red curves show the current upon tip retraction for graphene on SiO_2 and hBN, respectively. Again, we observed exponential decay for SiO_2, and an initial region of parabolic decay for hBN, indicative of the compression of the graphene towards the hBN substrate. The dotted lines show the tunneling current upon reapproach of the tip. The SiO_2 curves are nearly identical, while the hBN curves exhibit significant hysteresis, with the tunneling current offset to smaller values of Δz upon reapproach.

The large hysteresis suggests that the separation between the graphene and the tip depends on the direction of the tip motion. This provides further evidence that the tip is pushing and pulling the graphene, as there should be no significant source of hysteresis between a tip retraction and approach given an immobile graphene sheet. When the tip is only retracted a short distance such that the tunneling current remains finite (blue), the graphene remains stuck to the tip and consequently no hysteresis is observed. The inset of Supplementary Figure 3(a) shows a similar measurement on hBN taken with a very small initial tunneling current, such that the tip starts far from the graphene. In this case, the decay is much closer to exponential, with virtually no hysteresis.

Finally, we take similar retraction measurements on misaligned graphene on hBN (Supplementary Figure 3(b)). In this case, we see very similar behavior to the nearly-aligned samples, where the decay of the tunneling current is first parabolic before becoming exponential, and the decay becomes slower as the initial tip-sample separation is decreased (black to red to blue). This demonstrates that the ability to compress or decompress the graphene relative to the hBN is a property of the two materials independent of their relative rotation.

SUPPLEMENTARY NOTE 3 - LOCAL BAND GAPS

Recent transport experiments by multiple groups have demonstrated electronic band gaps in nearly-aligned graphene on hBN heterostructures [2–6]. Gap sizes as large as about 50 meV have been observed for devices in perfect alignment, and the gap size decreases as the misalignment is increased, a phenomenon not yet fully understood. The origin of the gaps themselves is still under debate, with proposals suggesting the gap either arises from sublattice symmetry breaking in the graphene [7, 8], through many-body interactions [9–11], or through a combination of the two.

It is natural, then, to expect to observe these band gaps in STS measurements of nearly-aligned devices, though to this point no such gaps have been reported [12]. In this study, we are also unable to unambiguously identify large band gaps in our dI/dV spectroscopy measurements. To test for such gaps, we take dI/dV as a function of back gate voltage, shown in Supplementary Figure 4. We may first look for signatures of a band gap in individual dI/dV curves, as shown in the inset of Supplementary Figure 4, taken at charge neutrality. However, the dI/dV shows a characteristic V-shape of an ungapped Dirac cone, rather than a gapped Dirac cone, in which the dI/dV should go to zero and exhibit a flat area in energy roughly equal to the magnitude of the gap. This limits the maximum size of the band gap to roughly the magnitude of our ac excitation bias, around 10 meV.

We may also examine the movement of the Dirac point in sample voltage (or energy) as a function of back gate voltage (or carrier density). In an ungapped Dirac cone, the movement of the Dirac point is expected to disperse as the square root of gate voltage [13]. In a gapped cone, there should either be a jump discontinuity in the position of the Dirac point, or a region of linear movement surrounding the Fermi energy (zero sample voltage), depending on the movement of the Fermi energy through the gap. However, we are never able to unambiguously identify a such a departure form the basic square root of gate voltage dispersion in any of our gate maps.

As a result, we have no evidence of band gaps anywhere near the size observed in global transport measurements. However, this is not totally unexpected. These devices never exhibit globally commensurate states, where a band gap of order 50 meV is expected simply from the potential difference of the two graphene sublattices [7, 8]. This instead suggests that while some small component of the band gap may emerge from weak sublattice symmetry breaking due to the small equilibrium graphene strain fields, it is likely enhanced significantly through many-body interactions. Unfortunately, our large metal tip, sitting around 1 nm or less away from the sample surface, likely screens out these many-body interactions, leaving a smaller band gap which we are not able to clearly resolve.

To attempt to circumvent this issue, we have taken gate maps in both extremes of the tip position. By acquiring the gate map with the tip very far away from the surface, we can attempt to minimize this screening. Alternatively, by moving the tip very close to the surface, we can attempt to increase the area of commensurate graphene on hBN (via the mechanism central to this work), and in the limit of the entire sample becoming commensurate, the

replica Dirac points should disappear and a large band gap may emerge without the need for significant many-body enhancement. However, in no case are we able to observe a band gap. Supplementary Figure 4 shows the result for a very close tip, and we still observe the replica Dirac points with no unambiguous band gap. This is also not unexpected, as we expect the tip can only modify the graphene lattice on length scales of about one moiré unit cell, which is not sufficient to localize electrons and open a gap (see Supplementary Note 5 for a theoretical analysis). The gate maps at very large tip separations look similar to the small separations, suggesting we are not able to move the tip far enough away to reduce possible screening effects in the incommensurate case. While our measurements are unable to directly address the nature of these band gaps, the lack of their observation points to a significant many-body contribution to their magnitude at equilibrium.

SUPPLEMENTARY NOTE 4 - ELASTIC THEORY FOR GRAPHENE/hBN MOIRÉ SUPERLATTICES

Geometric setup

Unstrained graphene deposited on hBN creates a moiré pattern controlled by the interlayer rotation angle θ and the lattice mismatch $\delta \approx 1.8\%$ between the crystals (graphene's lattice constant $a_0 \approx 0.246$ nm is smaller than hBN's $a'_0 \approx 0.250$ nm).

The graphene lattice is generated by the primitive vectors $\mathbf{a}_{1,2}$, of modulus a_0, which we write in a matrix form as $\hat{a} = (\mathbf{a}_1, \mathbf{a}_2)$ (the $\mathbf{a}_{1,2}$ are the *columns* of $\hat{a}$). The center of the graphene unit cells are at $\mathbf{r_n} = \hat{a}\mathbf{n}$. Likewise the unit cells of hBN are centred at $\mathbf{r'_n} = \hat{a}'\mathbf{n}$. The two Bravais bases are related by

$$\hat{a}' = \hat{R}\hat{a}, \qquad \text{(Supplementary Equation 2)}$$

where the rotation and scaling transformation $\hat{R}$ is

$$\hat{R} = (1+\delta)\begin{pmatrix} \cos\theta & -\sin\theta \\ \sin\theta & \cos\theta \end{pmatrix} \qquad \text{(Supplementary Equation 3)}$$

We assume that θ and δ are such that the two lattices are commensurate. This implies that there exists a finite moiré superlattice, with primitive vectors $\hat{A} = (\mathbf{A}_1, \mathbf{A}_2)$, such that

$$\hat{A} = \hat{a}\hat{N} = \hat{a}'\hat{N}', \qquad \text{(Supplementary Equation 4)}$$

where $\hat{N}$ and $\hat{N}'$ are *integer* 2×2 matrices. As an example, if $\theta = 0$ and $\delta = 1/55$, we have $\hat{N} = 56 \times \mathbb{1}$ and $\hat{N}' = 55 \times \mathbb{1}$. The period $L_{\mathrm{M}} = |\mathbf{A}_i|$ reads,

$$L_{\mathrm{M}} = \frac{1+\delta}{\sqrt{2 + (2+\delta)\delta - 2(1+\delta)\cos\theta}} a_0 \qquad \text{(Supplementary Equation 5)}$$

and is plotted in Supplementary Figure 5 as a function of θ.

The conjugate momenta of the two lattices are denoted by $\hat{g} = 2\pi\hat{a}^{-1}$ and $\hat{g}' = 2\pi\hat{a}'^{-1}$, while the momenta of the superlattice are

$$\hat{G} = 2\pi\hat{A}^{-1} = \hat{N}^{-1}\hat{g} = \hat{N}'^{-1}\hat{g}'. \qquad \text{(Supplementary Equation 6)}$$

Note that matrices $\hat{g}$, $\hat{g}'$ and $\hat{G}$ have the corresponding conjugate momenta ($\mathbf{g}_{1,2}$ etc.) as *rows*, not columns.

In general, we can always write $\hat{N}$ and $\hat{N}'$ in terms of $\hat{R}$. To do this we assume that in the unit cell there is a single moiré beating (it is a minimal cell). In this case $\hat{G} = \hat{g} - \hat{g}'$. This allows us to write

$$\begin{aligned} \hat{N} &= \hat{a}^{-1}(\mathbb{1} - \hat{R}^{-1})^{-1}\hat{a} \\ \hat{N}' &= \hat{a}^{-1}(\hat{R} - \mathbb{1})^{-1}\hat{a} \end{aligned} \qquad \text{(Supplementary Equation 7)}$$

which implies also

$$\begin{aligned} \hat{N}'^{-1}\hat{N} &= \hat{a}^{-1}\hat{R}\hat{a} = \hat{a}^{-1}\hat{a}' \\ \hat{N} - \hat{N}' &= \mathbb{1} \\ \hat{N}'\hat{N}^{-1} &= \hat{a}^{-1}\hat{R}^{-1}\hat{a} = \mathbb{1} - \hat{N}^{-1} \end{aligned} \qquad \text{(Supplementary Equation 8)}$$

(In all the expressions above, we may change $\hat{a}$ to $\hat{a}' = \hat{R}\hat{a}$ and they still hold.)

Potential created by the hBN substrate

The hBN substrate creates a van der Waals potential that attracts the graphene sample. This adhesion, however, depends on the local stacking across the moiré, and is strongest for carbon-boron Bernal alignment. Those parts of the moiré are thus more strongly bound to the substrate than the other regions. The spatially varying adhesion landscape makes

graphene deform elastically with a three-dimensional displacement field $\mathbf{u}(\mathbf{r}) = (u_x, u_y, h)$. This deformation expands the preferred carbon-on-boron regions, while other regions contract. To describe this elastic-adhesion interplay we first model the adhesion potential.

We will assume that the hBN substrate is a rigid crystal. Microscopic simulations [1] employing realistic carbon potentials have characterised the graphene/hBN adhesion energy per graphene unit cell for different perfect stackings as a function of interlayer distance z, i.e. $V_\alpha(z)$, where α = AA (aligned lattices), α = CB (carbon-on-boron), and α = CN (carbon-on-nitrogen) are the three different perfect stackings. The results are reproduced in Supplementary Figure 6. We find that these results can be accurately fitted by the following model

$$V_\alpha(z) = -\frac{5}{6} V_\alpha \left[\left(\frac{d_\alpha}{z} \right)^{11} - \frac{11}{5} \left(\frac{d_\alpha}{z} \right)^{5} \right], \qquad \text{(Supplementary Equation 9)}$$

where d_α and V_α represent the equilibrium distance and adhesion potential, respectively. The fit is shown in see Supplementary Figure 6, and the resulting fitting values (in Å and meV per graphene unit cell) are shown in Supplementary Table 1.

In addition to the adhesion energy V_α, the equilibrium interlayer distance d_α varies locally with the stacking configuration. In the absence of external perturbations, the graphene/hBN moiré pattern is thus spontaneously corrugated, with an out-of-plane displacement $h(\mathbf{r})$ relative to the $z = d_{\rm CB}$ plane, so that the interlayer distance across the sample

$$z(\mathbf{r}) = d_{\rm CB} + h(\mathbf{r}) \qquad \text{(Supplementary Equation 10)}$$

interpolates between the different d_α, see Supplementary Figure 7(a) and top (light) curve in Supplementary Figure 8(a). Here $\mathbf{r} = (x, y)$ is the position in the plane, with $\mathbf{r} = 0$ chosen at a carbon-boron stacking point. The out-of-plane corrugation has a maximum amplitude of around

$$h_{\rm corr} = \max h(\mathbf{r}) = d_{\rm AA} - d_{\rm CB} \approx 56 \text{ pm}. \qquad \text{(Supplementary Equation 11)}$$

We observe similar height fluctuations in our STM measurements, and numerous other groups have also observed these in AFM measurements as well [2, 14–17].

The adhesion potential $V_{\rm S}(\mathbf{r})$ is similarly modulated, see Supplementary Figure 7 and top (light) curve in Supplementary Figure 8(b). The adhesion energy of a graphene unit cell centred at $\mathbf{r}$ will be given by $V_{\rm S}(\mathbf{r})$, which interpolates between the different adhesion minima V_α. In this model we assume the adhesion potential (and also the equilibrium corrugation)

can be approximated by its six lowest harmonics (first star), namely $\pm\mathbf{g}'_{1,2,3}$, where we have defined the extra momentum $\mathbf{g}'_3 = -(\mathbf{g}'_1 + \mathbf{g}'_2)$. We encode this first star by dimensionless vectors

$$\boldsymbol{\nu}_1 = (1, 0),\ \boldsymbol{\nu}_2 = (0, 1),\ \boldsymbol{\nu}_3 = (-1, -1), \qquad \text{(Supplementary Equation 12)}$$

so that

$$V_\mathrm{S}(\mathbf{r}) = v_0 + \Re\left\{v_1 \sum_{i=1}^{3} e^{i\boldsymbol{\nu}_i \hat{g}'\mathbf{r}}\right\} \qquad \text{(Supplementary Equation 13)}$$

for some real ϕ_0 and complex ϕ. This specific form of the potential assumes that $V_\mathrm{S}(\mathbf{r})$ has an extremum at the center and corners $\mathbf{r}_\alpha$ of the hexagonal hBN unit cell. The potential at the three extrema $V_\alpha = V_\mathrm{S}(\mathbf{r}_\alpha)$ are encoded into v_0 and v_1,

$$\begin{aligned} v_0 &= (V_\mathrm{AA} + V_\mathrm{CN} + V_\mathrm{CB})/3 \\ v_1 &= -\frac{V_\mathrm{AA} + V_\mathrm{CN} - 2V_\mathrm{CB}}{9} - i\frac{V_\mathrm{AA} - V_\mathrm{CN}}{3\sqrt{3}} \end{aligned} \qquad \text{(Supplementary Equation 14)}$$

The corrugation $h(\mathbf{r})$ in Supplementary Figure 7(a) was built using this same procedure, with d_0 and d_1 defined as $v_{1,2}$ above, albeit with $d_\alpha - d_\mathrm{CB}$ in place of V_α,

$$h(\mathbf{r}) = d_0 + \Re\left\{d_1 \sum_{i=1}^{3} e^{i\boldsymbol{\nu}_i \hat{g}'\mathbf{r}}\right\}. \qquad \text{(Supplementary Equation 15)}$$

Consider now a perfectly flat and unstrained graphene sample at the plane $z = d_\mathrm{CB}$. The unstrained graphene cells are centred at

$$\mathbf{r}_\mathbf{n} = \hat{a}\mathbf{n}, \qquad \text{(Supplementary Equation 16)}$$

with an integer vector $\mathbf{n}$. We now consider a lattice distortion field

$$\mathbf{u}(\mathbf{r}) = (u_x, u_y, h) \qquad \text{(Supplementary Equation 17)}$$

The unit cells will be displaced to $\mathbf{R}_\mathbf{n} = \mathbf{r}_\mathbf{n} + \mathbf{u}(\mathbf{r}_\mathbf{n}) = \hat{a}\mathbf{n} + \mathbf{u}_\mathbf{n}$. The total adhesion energy in a supercell may be written as a sum over the set of graphene $\mathbf{n}$ vectors (a total of $\det\hat{N}$) that span the supercell

$$U_\mathrm{S} = \sum_{\mathbf{n}}^{\text{supercell}} V_\mathrm{S}(\mathbf{R}_\mathbf{n}) = \sum_{\mathbf{n}}^{\text{supercell}} \left(v_0 + \Re\, v_1 \sum_{i=1}^{3} e^{-i\boldsymbol{\nu}_i \hat{G}(\mathbf{r}_\mathbf{n} - \hat{A}\hat{a}'^{-1}\mathbf{u}_\mathbf{n})}\right)$$

(Supplementary Equation 18)

Here we have used $\mathbf{u_n} = \hat{a}'^{-1}\hat{a}'\mathbf{u_n}$ and $\hat{g}'\hat{a} = (\hat{g} - \hat{G})\hat{a} = 2\pi - \hat{G}\hat{a}$, (recall that $\hat{G} = \hat{g} - \hat{g}'$). We have also used $\hat{g}'\hat{a}' = 2\pi$ and the fact that $\exp(i2\pi\boldsymbol{\nu}_i\mathbf{n}) = 1$, since $\boldsymbol{\nu}_i$ and $\mathbf{n}$ are both integer vectors. The expression for U_S above can be recast into an integral form at small angles, when $\det \hat{N}$ is large, since the terms become smooth in $\mathbf{n}$. Instead of $\sum_\mathbf{n}^\mathrm{supercell}$ one may do an integral $\frac{1}{\det A}\int_\mathrm{supercell} d^2r$. This also allows one to rediscretize the U_S sum with *any* mesh that covers the supercell, even one that is much coarser than the atomic mesh, for example $\mathbf{r_n} = \hat{b}\mathbf{n}$, with $\hat{b} = \hat{A}/m$, with m a small integer, e.g. $m = 4$ or 5. When rediscretizing, one should be careful to normalize the sum by the Jacobian $\det \hat{b}/\det \hat{a}$.

In practice this rediscretization works well because the deformation fields $\mathbf{u}(\mathbf{r})$ that result from this model are smooth on the moiré lengthscale L_M, so one needs only a few (m) points to within one L_M to accurately describe the deformation.

Elastic energy

The elastic energy U_E per supercell of a graphene deformation $\mathbf{u}(\mathbf{r})$ that is smooth on the atomic spacing is given by continuum elasticity theory,

$$U_\mathrm{E} = \frac{1}{2}\int_{\hat{A}} d^2r \left[2\mu \,\mathrm{Tr}(\hat{u}^2) + \lambda \,(\mathrm{Tr}\,\hat{u})^2\right], \qquad \text{(Supplementary Equation 19)}$$

Here $\hat{u} = u_{ij} = \frac{1}{2}(\partial_i u_j + \partial_j u_i + \partial_i h \partial_j h)$ is the strain, and $\lambda \approx 3.5$ eV/^{2} and $\mu \approx 7.8$ eV/^{2} are the Lamé factors for graphene. In the following, the quartic in h contribution to U_E will be neglected, since it is of the order of $(2h_\mathrm{corr}/L_\mathrm{M})^2 \approx 10^{-5}$. This approximation decouples the equilibrium corrugation from in-plane strains, so that $h(\mathbf{r})$ is always given by Supplementary Equation 15.

To evaluate U_E, one needs to approximate the derivatives $\partial_i u_j$ in $\hat{u}$ by finite differences in the two dimensional mesh $\mathbf{r_n} = \hat{b}\mathbf{n}$. Since this mesh is triangular in this case, the finite differences are best evaluated at the center of each triangle, i.e. in the dual honeycomb lattice formed by all the triangle centers. When thus evaluating the integral as a discrete sum of finite differences, care must be taken once more to properly normalize to the total supercell area divided by the number of dual mesh points (two) per mesh unit cell

$$U_\mathrm{E} = \frac{\det \hat{b}}{2}\sum_\mathbf{n}^\text{dual mesh} \frac{1}{2}\left[2\mu \,\mathrm{Tr}(\hat{u}_\mathbf{n}^2) + \lambda \,(\mathrm{Tr}\,\hat{u}_\mathbf{n})^2\right] \qquad \text{(Supplementary Equation 20)}$$

Equilibrium strains under uniform pressure

The (tensile) strain profile of the graphene sample at equilibrium is defined by the field

$$\frac{1}{2}\text{Tr}\hat{u}(\mathbf{r}) = \frac{a}{a_0} - 1 \qquad \text{(Supplementary Equation 21)}$$

where a is the average lattice constant of the sample at point $\mathbf{r}$. This strain profile arises in the sample at equilibrium as a result of the forces derived from the total elastic plus adhesion potential $U = U_\text{E} + U_\text{S}$. The expected strain using our model for U is shown in Supplementary Figure 8(c), with a cut along the white line shown in Supplementary Figure 8(e), top (light) curve. This strain is computed by minimizing $U(\mathbf{u_n})$ as a function of the discretized in-plane disortion field $\mathbf{u}(\mathbf{r_n}) = \mathbf{u_n}$, while $h(\mathbf{r})$ is fixed by the equilibrium value, Supplementary Equation 15, see Supplementary Figure 7(a) and Supplementary Figure 8(a), top (light) curve. The numerical optimization of U over in plane distortions $\mathbf{u_n}$ is efficiently implemented using the conjugate gradient method. The fact that the continuum approximation, Supplementary Equation 18, of the adhesion potential allows for a discretization mesh of the disortion field $\mathbf{u_n}$ that is conveniently coarser than the atomic spacing, allows for important numerical efficiency gains.

We see in Supplementary Figure 8(c) that CB-stacked regions are slightly expanded relative to graphene in vacuum, while AA, and to less extent also CN, are correspondingly compressed. Since this minimization assumes no global expansion of the sample in response to the adhesion, the spatial integral of $\frac{1}{2}\text{Tr}\hat{u}(\mathbf{r})$ is zero. The variation of strain is however rather small, around -0.2% at AA, $+0.3\%$ at CB.

The magnitude of this modulation is controlled by the adhesion energy of favoured CB regions relative to unfavourable AA regions, see top (light) curve of Supplementary Figure 8(b). It is reasonable to expect that enhancing this adhesion difference one could also enhance the spontaneous strain modulations. This is in principle simple to do. If we apply uniform hydrostatic pressure to the sample, graphene is pushed towards the substrate, which should enhance the adhesion difference, given the adhesion curves $V_\alpha(z)$ in Supplementary Figure 6. To confirm this, we add one more term $V_\text{P}(z) = P(z - d_\text{CB}) \det \hat{a}$ (the chosen z origin is arbitrary) to the substrate adhesion potentials $V_\alpha(z)$ in Supplementary Equation 9, where P is pressure. One then performs the minimization of $V_\alpha(z) + V_\text{P}(z)$, to find the modified values of V_α and d_α as a function of pressure P. As expected, the d_α decrease and $|V_\text{CB} - V_\text{AA}|$ is strongly enhanced. This is shown in Supplementary Figure 8(a) and (b) in

the range $P = 0$ to $P = 40$ GPa. The resulting equilibrium strain under pressure is shown in Supplementary Figure 8(d) and (e). They show a positive pressure-induced expansion at CB stacking regions that saturate for high pressures at the commensurate limit $a/a_0 = 1+\delta$, i.e. a 1.8% tensile strain so that graphene becomes locally commensurate to the underlying hBN crystal. The boundaries between CB regions become narrow and strongly compressed, reaching a tensile strain of $\sim -4\%$ at $P = 40$ GPa. The total energy per supercell of the sample

In all the above simulations the area of the sample is kept constant as pressure is increased. In particular, we did not allow up to now for the possibility of the sample developing a global uniform expansion $a/a_0 = 1 + \delta$ to conform to the substrate everywhere. While this configuration is indeed not energetically favorable under zero pressure, one can expect that at high-enough pressures, the enhanced adhesion differences that lead to expanded CB regions discussed above would also favor a globally commensurate phase. To evaluate the possibility of a structural transition into said phase, we compute its energy as a function of pressure, relative to that of graphene in vacuum ($U = 0$). The energy per unit cell for the globally commensurate phase reads

$$V_{\mathrm{CB}}(P) + 2\det\hat{a}(\lambda+\mu)\delta^2 \qquad \text{(Supplementary Equation 22)}$$

and is shown as a function of pressure by the dashed line in Supplementary Figure 8(f). It indeed becomes smaller than the energy of the sample with the moiré strain profile (solid line) for pressure above a critical value $P_{\mathrm{c}} = 0.15GPa$. We thus expect that as soon as pressure exceeds P_{c}, the graphene sample would undergo a (first-order) structural transition into global commensuration. Electronically, this phase is expected to develop a large gap at the Dirac point around $50 - 200$ meV [7, 10, 18].

An alternative method to hydrostatic pressure to enhance moiré strains is to apply pressure with a metallic plate. Graphene adheres to most metals more strongly than to hBN, so that a perfectly flat metallic plate at constant $z(\mathbf{r}) = d_{\mathrm{CB}}+h_0$ pressing onto the sample would completely suppress sample corrugations, so that $h(\mathbf{r}) \approx h_0$ (we have incorporated the equilibrium plate-graphene distance into h_0 here). As a result, V_α would not correspond to the minimum of $V_\alpha(z)$ but rather to $V_\alpha(d_{\mathrm{CB}}+h_0)$ at constant the z. Supplementary Figure 9(a) shows the corresponding $V_{\mathrm{S}}(\mathbf{r})$ for different values of h_0 along a $CB - CN - AA - CB$ spatial path. The equilibrium strains for $h_0 = -0.5$Å are shown in panel (b). Note the

expanded CB regions, and the narrow boundaries, analogous to the case of $P = 40$ GPa hydrostatic pressure, with a similar range of tensile strains from 1.8% (CB) to $\sim -4\%$ (AA). Panel (c) shows the total energy as a function of h_0, both for the incommensurate and the globally commensurate phase. All energies are taken relative to that of graphene in vacuum ($h_0 \to \infty$, i.e. $U = 0$). We see that, as long as the constraint $z_0 = d_{\rm CB} + h_0$ is uniform across the sample, graphene is expected to spontaneously snap into a globally commensurate phase for $h_0 \lesssim -0.12$Å. As for the case of hydrostatic pressure, the transition is first order, and is expected to be thermally activated.

It is also interesting to note that the total energy of the incommensurate phase has a minimum at a value of h_0 around

$$h_{\rm min} = 0.37 \text{ Å}, \qquad \text{(Supplementary Equation 23)}$$

somewhat smaller than the corrugation $h_{\rm corr}$ in the equilibrium sample, which implies that within the commensurate phase, a repulsive (attractive) force will develop below (above) this position between graphene and the metallic plate.

Tip potential

The effect of a large metallic tip close to the sample is similar to the above analysis of pressure by a metallic plate. In the case of a metallic tip, h_0 should be taken to be position dependent. For a paraboloid-like tip of radius $R_{\rm tip}$ with its apex at (x_0, y_0, h_0), we have

$$h_{\rm tip}(\mathbf{r}) = h_0 + \frac{(x - x_0)^2 + (y - y_0)^2}{2R_{\rm tip}} \qquad \text{(Supplementary Equation 24)}$$

Typical tip radii, around $R_{\rm tip} \approx 200$ nm, are quite large compared to the moiré lengthscale $L_{\rm M} \lesssim 14$ nm, which justifies the above paraboloid model. Graphene does not conform to the tip at all positions, however, since it is constrained by boundary conditions to remain stuck to hBN far from the tip (this precludes a tip-induced transition into a globally commensurate phase). The tip constraint should therefore be truncated to values of h below a maximum retraction value $h_{\rm max}$, so that

$$h(\mathbf{r}) = \begin{cases} h_{\rm tip}(\mathbf{r}) \text{ if } h_{\rm tip}(\mathbf{r}) < h_{\rm max} \\ h_{\rm eq}(\mathbf{r}) \text{ otherwise} \end{cases} \qquad \text{(Supplementary Equation 25)}$$

This profile is illustrated in Fig. 1(c) of the main text. The value of $h_{\max}$ can be estimated to be $h_{\max} \approx 1-2$ Å from the crossover regime in Fig. 2(b) of the main text. The interaction range of the tip, i.e. the distance $R_{\max}$ from its apex below which the sample/substrate adhesion is controlled by the tip, reads, for tip height $h_0 < h_{\max}$,

$$R_{\max} = \sqrt{2R_{\mathrm{tip}}(h_{\max} - h_0)} \qquad \text{(Supplementary Equation 26)}$$

If $h_0 > h_{\max}$, the sample is not adhered to the tip, and we assume $R_{\max} = 0$.

As revealed by the tip retraction experiment of the main text, the sample $h(\mathbf{r})$ does not exactly conform to the tip $h_{\mathrm{tip}}(\mathbf{r})$, and it furthermore recedes from the tip less abruptly as h_0 exceeds $h_{\max}$, but the above model proves to be rather accurate to describe the mechanical tip-sample interactions.

The influence of the tip on the sample strain throughout a scan can be incorporated rather economically into the adhesion potential by spatially modulating the value of v_0 v_1,

$$U_{\mathrm{S}} = \frac{\det \hat{b}}{\det \hat{a}} \sum_{\mathbf{n}}^{\text{supercell}} \left(v_0(\mathbf{r_n}) + \Re\, v_1(\mathbf{r_n}) \sum_{i=1}^{3} e^{-i\boldsymbol{\nu}_i \hat{G}(\mathbf{r_n} - \hat{A}\hat{a}'^{-1}\mathbf{u_n})} \right)$$

(Supplementary Equation 27)

where $v_0(\mathbf{r}), v_1(\mathbf{r})$ are defined as in Supplementary Equation 14, but with $V_\alpha(d_{\mathrm{CB}} + h(\mathbf{r}))$ from Supplementary Equation 9 and Supplementary Equation 25 in place of constants V_α.

Scanning tip and dynamical strain

A modulation of the graphene/hBN distance $z(\mathbf{r}) = d_{\mathrm{CB}} + h(\mathbf{r})$ imposed by an STM tip produces a spatial variation in the total energy density accumulated in the deformation field. The total energy stored in the deformation field has a minimum at $h_{\min} = 0.37$Å. A gradient in $h(\mathbf{r})$ will therefore give rise to forces that will tend to push the moiré strain profile, and in particular the boundaries between locally commensurate CB regions, towards points with $h(\mathbf{r}) = h_{\min}$. Effectively, therefore, a tip at a height $h_0 < h_{\min}$ will tend to push stacking boundaries away from its apex, while if $h_0 > h_{\mathrm{corr}}$ the tip will appear to attract stacking boundaries. These forces dynamically modify the deformation field as the STM tip scans the sample. In this section we analyze this dynamical response, and its consequences for the apparent strain measured by the tip.

In the preceding section, the total energy per supercell $U = U_{\mathrm{E}} + U_{\mathrm{S}}$ in the presence of a tip has been expressed as a function of distortions $\mathbf{u_n}$ and rotation angle θ on a conveniently coarse discretization of the moiré pattern. All parameters of the model are known to reasonable precision, including the tip radius $\mathbf{R}_{\mathrm{tip}}$ and the maximum retraction distance h_{max}. It is then possible to minimize the total energy numerically to obtain the equilibrium elastic configuration for each tip position as it scans the sample. As in the cases of hydrostatic and plate pressure, this is done using conjugate gradient methods. The result is a discretisation of the equilibrium deformation $\mathbf{u}(\mathbf{r}, \mathbf{r}_0, h_0)$, and the associated strain tensor $\hat{u}(\mathbf{r}, \mathbf{r}_0, h_0)$, as a function of tip position $\mathbf{r}_0 = (x_0, y_0, h_0)$.

When relaxing the lattice in response to a scanning tip, it is important to choose as seed to the conjugate gradient method the relaxed configuration from the prior tip position. This choice is irrelevant for larger values of h_0, since there is no scanning hysteresis. For smaller h_0, however, the tip hysteretically drags the stacking domains along, so the choice of seed is important, as opposite scanning directions yield different configuration paths.

Due to the $\mathbf{r}_0$ dependence of $\hat{u}$, the graphene tensile strain $a/a_0 - 1$ (normalized change in the average lattice constant) as measured by the tip (what we dub here 'dynamical strain') is not simply the static expansion $\frac{1}{2}\mathrm{Tr}\hat{u} = \frac{1}{2}(\partial_x u_x + \partial_y u_y)$. It also acquires a dynamical contribution. The dynamical strain reads

$$\frac{a}{a_0} - 1 = \frac{1}{2}\left(\partial_x u_x + \partial_y u_y + \partial_{x_0} u_x + \partial_{y_0} u_y\right)\big|_{\mathbf{r}_0 = \mathbf{r}} \qquad \text{(Supplementary Equation 28)}$$

We have simulated this dynamical strain for a $\theta = 0^\circ$ sample, scanned with an STM tip of realistic radius $R_{\mathrm{tip}} = 200$ nm, and a $h_{\mathrm{max}} = 1.5$ Å. The results for varying tip-sample scanning distances h_0 are shown in 10. Panel (a) shows the static expansion, corresponding to a tip with negligible interaction with the sample, $h_0 \to \infty$ (actually $h_0 > h_{\mathrm{max}}$ in our simplified adhesion model). As in Supplementary Figure 8(c), it has smooth strain profiles in the $\sim -0.2\%$ to 0.3% range, with CB regions expanded relative to the rest. The dynamical strain as the tip scans at $h_0 < h_{\mathrm{max}}$ shows three distinct regimes, which we describe below.

The 'attractive regime', panels (b)-(e), corresponds to $h_{\mathrm{min}} < h_0 < h_{\mathrm{max}}$, see Supplementary Equation 23. In this scanning range the tip locally lifts the sample away from the substrate, irrespective of its position $\mathbf{r}_0$. Consequently, adhesion differences $|V_{\mathrm{CB}} - V_{\mathrm{AA}}|$ are reduced under the tip, but remain the same away from the tip. This produces an effective attraction between the tip and the surrounding the stacking boundaries. As a result, the

boundaries are partially dragged along by the tip as it scans, and therefore appear to be expanded (positive dynamical strain). The CB regions, in contrast, exhibit negative dynamical strain (they appear compressed). The latter is a consequence of a basic property of the dynamical strain. Just like the static strain of an asymptotically relaxed sample, the dynamical strain integrates to zero across the sample, so that a positive dynamical strain of stacking boundaries should be compensated by a negative dynamical strain elsewhere. (This is satisfied as long as the sample as a whole doesn't slide in response to the scanning tip, and that the dynamical strain is not discontinuous, i.e it is non-hysteretic).

A crossover pattern is obtained at $h_0 \approx h_{\rm min}$, panel (f), before entering a 'repulsive regime' for $h_0 < h_{\rm min}$. In this scanning range, the tip pushes stacking boundaries away as it moves. As the sample as a whole is assumed to not slide as a result of scanning, the boundaries quickly slide back under the tip when they are pushed beyond a maximum distance. This quick sliding makes them appear compressed far above their equilibrium compression $\sim -0.2\%$. The resulting dynamical strain is shown in panels (g)-(i). If $h_0 \gtrsim 0$ this pushing is non-hysteretic (panels (g) and (h)), so that opposite scanning directions follow the same path in the sample configuration space. The dynamical strain maps preserve all the moiré symmetries. If h_0 is decreased below zero, however, (the tip is pushing the sample strongly enough into the substrate), the area of commensurate CB regions grow further, their static strain approaches the maximum static value $a/a_0 - 1 = \delta$ as in Supplementary Figure 9(b) (the dynamical strain is not bounded), and the stacking boundaries are vigorously pushed away from the tip. When boundaries are pushed far enough in this regime, they snap back irreversibly under the tip [19], which gives a discontinuous jump in the dynamical strain. This is a 'hysteretic repulsive regime'. The corresponding dynamical strain map (panel (i)) then depends on the scanning direction and breaks the C_3 symmetry of the moiré.

All these regimes are observed in our experiments, and closely match the simulations above, with one exception. Deep in the hysteretic repulsive regime, the observed dynamical strain may develop one further transition not observed in the simulations, whereby the dynamically compressed stacking boundaries suddenly switch to a positive dynamical strain, well above the maximum static value $\delta = 1.8\%$. This can be understood as the result of reversible sample delamination in front of the tip. The compression accumulated in a pushed boundary can exceed a value where the sample becomes unstable to buckling out of plane in front of the tip. This structural transition greatly relaxes the accumulated compression,

which makes the stacking boundary recede further away from the tip, and thus appear to develop a local expansion. The possibility of delamination is not included in our simulations, however, so we can only argue about it on a qualitative level.

Supplementary Movies 1-4 provide animations of the dynamical and instantaneous strain under the scanning tip in the different response regimes. Representative snapshots of these simulations are shown in Supplementary Figure 11.

SUPPLEMENTARY NOTE 5 - LOCAL DENSITY OF STATES UNDER THE TIP

The pressure-induced commensuration between graphene and the hBN substrate has powerful implications for the electronic structure. Assuming a hopping amplitude $t_\perp \approx 0.3$ eV between carbon and boron atoms, and a hBN valence band at $\epsilon_{\rm B} \approx 1.4$ eV with respect to graphene's Dirac point, the graphene regions with CB-stacking will acquire a substrate induced self-energy that is different in the two sublatices, $\Sigma_{\rm A} \approx t_\perp^2/\epsilon_{\rm B}$, $\Sigma_{\rm B} = 0$. This sublattice imbalance $\Delta = \Sigma_{\rm A} - \Sigma_B \approx 64$ meV takes the form of a mass term $\frac{1}{2}\Delta\sigma_3$ in graphene's Dirac spectrum (plus an unimportant scalar term of equal magnitude). A spatially uniform mass term is expected to open a gap Δ in graphene's Dirac spectrum. In our samples, however, the pressure-induced CB commensuration is confined to within one moiré period approximately, $\lambda_{\rm CB} \approx L_{\rm M} \approx 14$nm, according to our simulations (see red area in Fig. 1d of main text). A non-straightforward question is whether this area is enough to induce a gap in the local density of states (LDOS) that could be measured by the tip.

A qualitative argument can be used to answer this question. A gap of magnitude Δ corresponds to the localization of states of typical wavelength $\lambda_\Delta = hv_{\rm F}/\frac{\Delta}{2} \approx 165$ nm. Therefore, the minimum spatial extension required of the pressure-induced CB stacking should be of the same order, $\lambda_{\rm CB} \gtrsim \lambda_\Delta$, hence much larger than the actual area affected by our tips.

We have also performed quantitative simulations of the LDOS under the tip, with the graphene lattice subjected to the tip-induced strains obtained with our elastic model. The local registry between graphene and the hBN substrate create a sublattice- and position-dependent self-energy, while in-plane strains also induce pseudogauge fields [18, 20]. The LDOS calculation is performed using the Kernel Polynomial method [21], using the Jackson kernel, and a polynomial order $N = 6000$ (N determines the energy resolution of

the method). Note that no many-body effect [9–11] are included here. The computed LDOS is shown in Supplementary Figure 12 for three different values of the the tip height $h_0 = -1.0$ Å, -0.5 Å and ∞. For comparison we also include the result with a flat plate at height $h_0 = -1.0$ Å, like the one described in Supplementary Figure 9, which results in a periodic mesh of thin solitons throughout the sample when artificially precluding a globally commensurate transition. Dashed lines in the zoom (panel b) correspond to fits to a gapped Dirac spectrum of the form $\mathrm{LDOS}(\epsilon) \propto \mathrm{Re}\sqrt{(\tilde{\Delta}/2)^2 - (\epsilon - \epsilon_0)^2}$, where $\tilde{\Delta}$ is an estimate of the gap that would be obtained for polynomial expansion order $N \to \infty$. Finally, we also include the results of a fully commensurate graphene sheet.

We see that, regardless of the height of the tip, the LDOS around neutrality is the same, and the LDOS gap $\tilde{\Delta}$ derived from the fit is zero to within less than a millielectronvolt. This is consistent with the qualitative argument above, and with our experimental observations, which do not resolve an LDOS gap regardless of tip pressure. For the plate, however, which induces CB stacking throughout a significant fraction of the sample area, we numerically obtain a finite LDOS gap $\tilde{\Delta} \approx 16$meV. The ideal value $\tilde{\Delta} = \Delta$ would be achieved only in the globally commensurate phase (black curves in Supplementary Figure 12), which is expected to be the true ground state for a plate with $h_0 < -0.12$ Å, as shown in Supplementary Figure 9(c).

SUPPLEMENTARY NOTE 6 - COMPARISON TO RESULTS OF WOODS *et al.*

In a recent work, Woods *et al.* analyzed the morphology, as measured with an AFM tip, of graphene/hBN heterostructures as a function of relative twist angle θ between the two crystals [2]. The main observation was a change in the characteristic width (FWHM) of boundaries between carbon-boron stacking domains. At angles above 1°, said width appeared to be equal to half a moiré period $L_\mathrm{M}(\theta)/2$, with L_M given in Supplementary Equation 5. This is the defining feature of a floating phase, where substrate-induced deformations of graphene are negligible. Below $\theta \approx 1°$, however, the observed width seemed to abruptly transition to a small fraction of L_M, around FWHM$= 0.2L_\mathrm{M}$, as would correspond to strong deformations of graphene due to the substrate. This abrupt jump was attributed to an incommensurate-to-commensurate structural transition, in the language of the Frenkel-Kontorova model [22].

Our own observation of the apparent topography and strain of small-angle samples, as measured by an STM tip at small tip separations, closely resembles the observations of Ref. [2] for their $\theta < 1^\circ$ samples marked by sharp moiré walls. Our interpretation, however, is very different from the one proposed by Woods *et al.* Simply by moving the tip away from the sample and repeating the topography measurement, we observe a transition to a smooth moiré profile. This clearly indicates that, at least in our samples, the Woods *et al.* interpretation of sharp sample boundaries produced by an equilibrium structural transition of the sample is not valid. The change in apparent strain maps with tip separation demonstrates that our tip is strongly invasive, and is itself the cause of the observed strain profiles of the sample. This is supported by our theoretical modelling, moreover, which suggests that the sample, when not affected by the tip, should not experience a detectable commensurate-incommensurate transition as a function of angle, and is instead almost perfectly smooth (FWHM $\approx 0.5L_\mathrm{M}$, floating phase) for angles down to $\theta = 0^\circ$.

To assess whether the apparent transition observed Woods *et al.* could be explained by the influence of the scanning tip as in our experiment, we have simulated, using the theory of Sect. , the FWHM of stacking boundaries as a function of angle $\theta = 0^\circ \ldots 3^\circ$ as measured by a metallic tip. The tip is assumed to scan at $h_0 = 0.1$ Å from the sample at all angles, so that at $\theta = 0^\circ$ it is in the repulsive, although non-hysteretic regime, with narrow boundaries and a FWHM around $0.1L_\mathrm{M}$. A sharp increase of the FWHM of stacking boundaries is indeed observed as the twist angle is increased above a certain threshold (just under 1° for this tip separation). The change in FWHM across the angle threshold is quite fast, but it is not discontinuous. The precise value of the threshold depends on geometric tip parameters R_tip and h_max, and corresponds to a moiré period approximately equal to the interaction range of the tip (Supplementary Equation 26), $R_\mathrm{max} \sim L_\mathrm{M}(\theta)$. Slightly adjusting the tip parameter h_max to 2.5 Å to tune this condition to $\theta \approx 1^\circ$ as in Woods *et al.*, we obtain results compatible with their observations (Supplementary Figure 13). This simulation suggests that our interpretation that sharp stacking boundaries at small angles are a tip-induced artifact, not an equilibrium property of the system, could also be relevant for the other scanning probe microscopy measurements of these systems.

SUPPLEMENTARY REFERENCES

[1] van Wijk, M. M., Schuring, A., Katsnelson, M. I. & Fasolino, A. Moiré patterns as a probe of interplanar interactions for graphene on h-BN. *Physical Review Letters* **113**, 135504 (2014).

[2] Woods, C. R. *et al.* Commensurate-incommensurate transition in graphene on hexagonal boron nitride. *Nature Physics* **10**, 451-456 (2014).

[3] Hunt, B. *et al.* Massive dirac fermions and hofstadter butterfly in a van der waals heterostructure. *Science* **340**, 1427-1430 (2013).

[4] Chen, Z.-G. *et al.* Observation of an intrinsic bandgap and landau level renormalization in graphene/boron-nitride heterostructures. *Nature Communications* **5**, 4461 (2013).

[5] Gorbachev, R. V. *et al.* Detecting topological currents in graphene superlattices. *Science* **346**, 448-451 (2014).

[6] Wang, L. *et al.* Evidence for a fractional fractal quantum hall effect in graphene superlattices. *Science* **350**, 1231-1234 (2015).

[7] Giovannetti, G., Khomyakov, P. A., Brocks, G., Kelly, P. J. & van der Brink, J. Substrate-induced band gap in graphene on hexagonal boron nitride: *Ab initio* density functional calculations. *Physical Review B* **76**, 073103 (2007).

[8] Sławińska, J., Zasada, I. & Klusek, Z. Energy gap tuning in graphene on hexagonal boron nitride bilayer system. *Phys. Rev. B* **81**, 155433 (2010).

[9] Song, J. C. W., Shytov, A. V. & Levitov, L. S. Electron interactions and gap opening in graphene superlattices. *Physical Review Letters* **111**, 266801 (2013).

[10] Bokdam, M., Amlaki, T., Brocks, G. & Kelly, P. J. Band gaps in incommensurable graphene on hexagonal boron nitride. *Physical Review B* **89**, 201404(R) (2014).

[11] Slotman, G. *et al.* Effect of structural relaxation on the electronic structure of graphene on hexagonal boron nitride. *Physical Review Letters* **115**, 186801 (2015).

[12] Yankowitz, M., Xue, J. & LeRoy, B. J. Graphene on hexagonal boron nitride. *Journal of Physics: Condensed Matter* **26**, 303201 (2014).

[13] Zhang, Y. *et al.* Giant phonon-induced conductance in scanning tunnelling spectroscopy of gate-tunable graphene. *Nature Physics* **4**, 627-630 (2008).

[14] Dean, C. R. *et al.* Hofstadter's butterfly and the fractal quantum hall effect in moire superlattices. *Nature* **497**, 598-602 (2013).

[15] Yang, W. *et al.* Epitaxial growth of single-domain graphene on hexagonal boron nitride. *Naturue Materials* **12**, 792-797 (2013).

[16] Tang, S. *et al.* Precisely aligned graphene grown on hexagonal boron nitride by catalyst free chemical vapor deposition. *Scientific Reports* **3**, 2666 (2013).

[17] Gallagher, P. *et al.* Switchable friction enabled by nanoscale self-assembly on graphene. *Nature Communications* **7**, 10745 (2016).

[18] San-Jose, P., Gutiérrez-Rubio, A., Sturla, M. & Guinea, F. Electronic structure of spontaneously strained graphene on hexagonal boron nitride. *Physical Review B* **90**, 115152 (2014).

[19] Yankowitz, M. *et al.* Electric field control of soliton motion and stacking in trilayer graphene. *Nature Materials* **13**, 786-789 (2014).

[20] San-Jose, P., Gutiérrez-Rubio, A., Sturla, M. & Guinea, F. Spontaneous strains and gap in graphene on boron nitride. *Physical Review B* **90**, 075428 (2014).

[21] Weiße, A., Wellein, G., Alvermann, A. & Fehske, H. The kernel polynomial method. *Rev. Mod. Phys.* **78**, 275-306 (2006).

[22] Chaikin, P. M. & Lubensky, T. C. *Principles of Condensed Matter Physics*, vol. 1 (Cambridge University Press, 2000).
