# Supplementary figures and images for: Pressure-induced commensurate stacking of graphene on boron nitride
Source: Nat Commun. 2016 Oct 20;7:13168. doi: 10.1038/ncomms13168 (PMC5462001; doi:10.1038/ncomms13168)

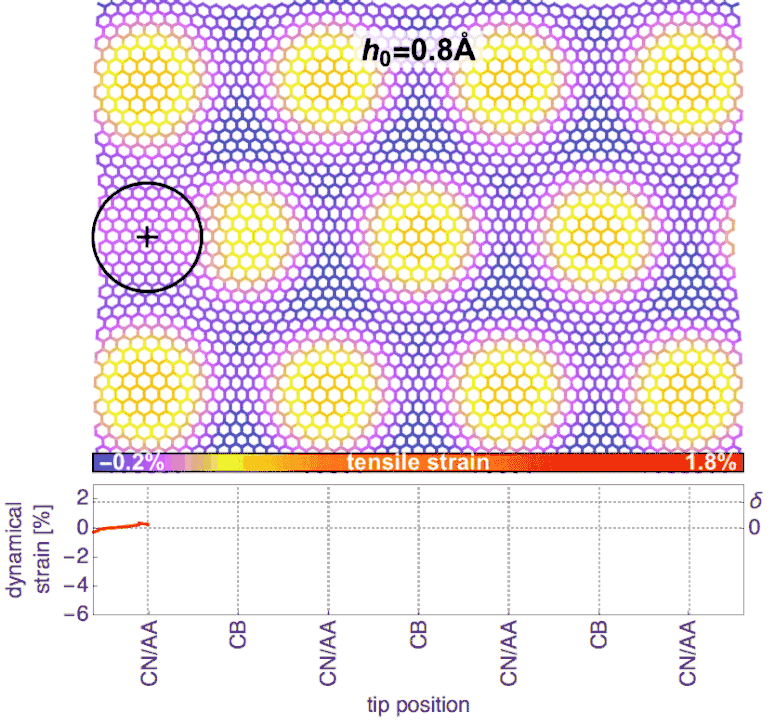

Supplement: Supplementary Movie 1 — Movement of the scanning probe tip across the moiré at 0.8 Angstrom above the equilibrium graphene position. [file ncomms13168-s2.gif]

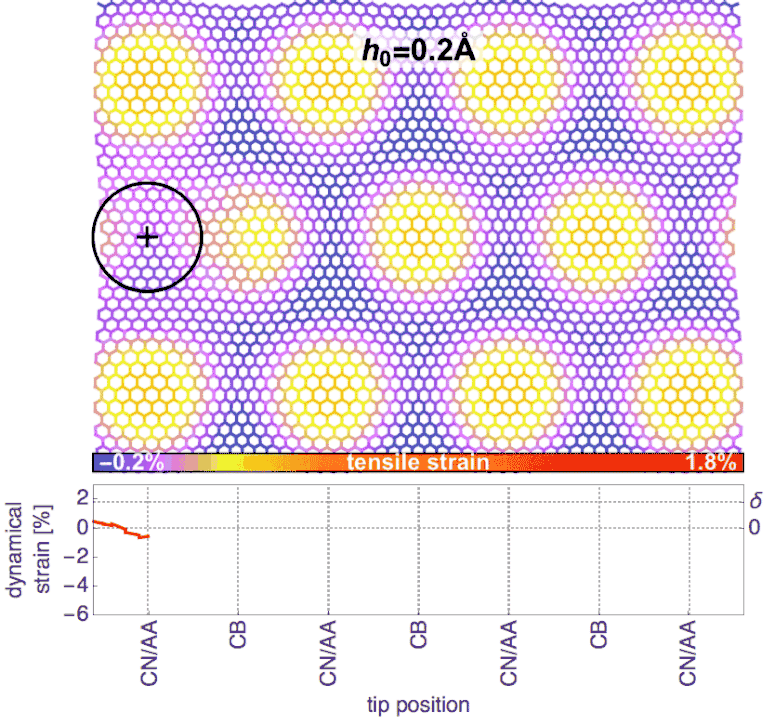

Supplement: Supplementary Movie 2 — Movement of the scanning probe tip across the moiré at 0.2 Angstrom above the equilibrium graphene position. [file ncomms13168-s3.gif]

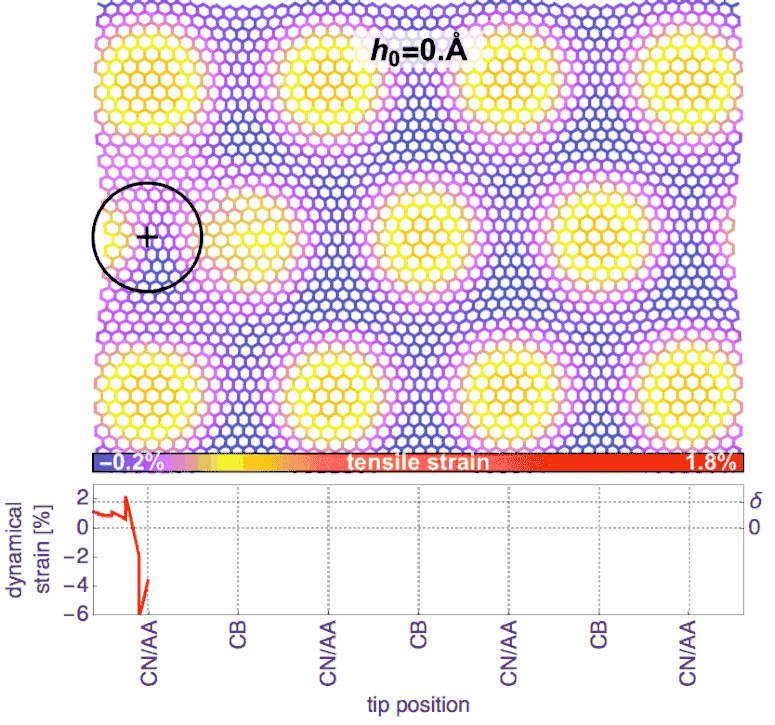

Supplement: Supplementary Movie 3 — Movement of the scanning probe tip across the moiré at the equilibrium graphene position. [file ncomms13168-s4.gif]

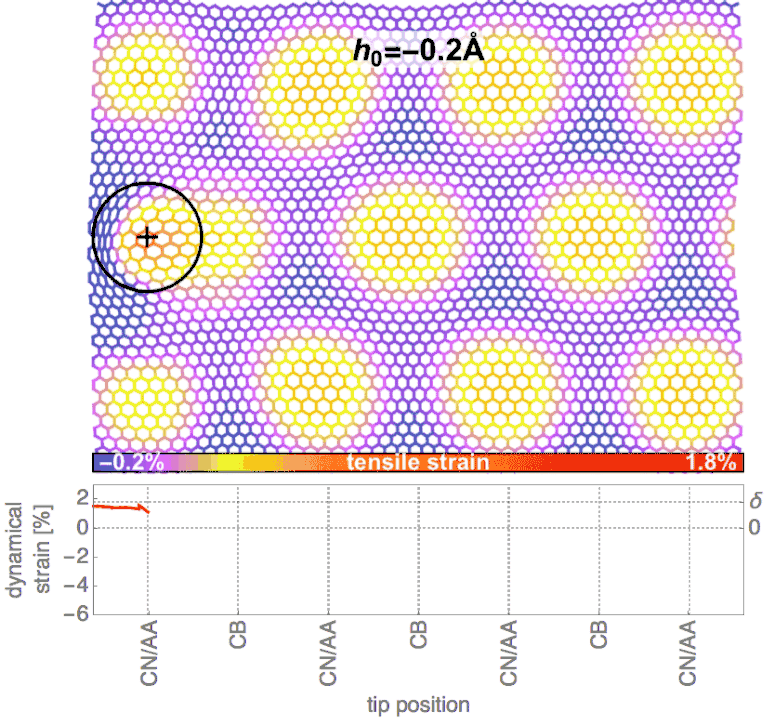

Supplement: Supplementary Movie 4 — Movement of the scanning probe tip across the moiré at -0.2 Angstrom above the equilibrium graphene position. [file ncomms13168-s5.gif]
